# Supplementary material for: Disruption of T-box transcription factor eomesa results in abnormal development of median fins in Oujiang color common carp Cyprinus carpio
Source: PLoS One. 2023 Mar 2;18(3):e0281297. doi: 10.1371/journal.pone.0281297 (PMC9980737; doi:10.1371/journal.pone.0281297)
Supplement: S1 Fig — (DOCX) [file pone.0281297.s001.docx]

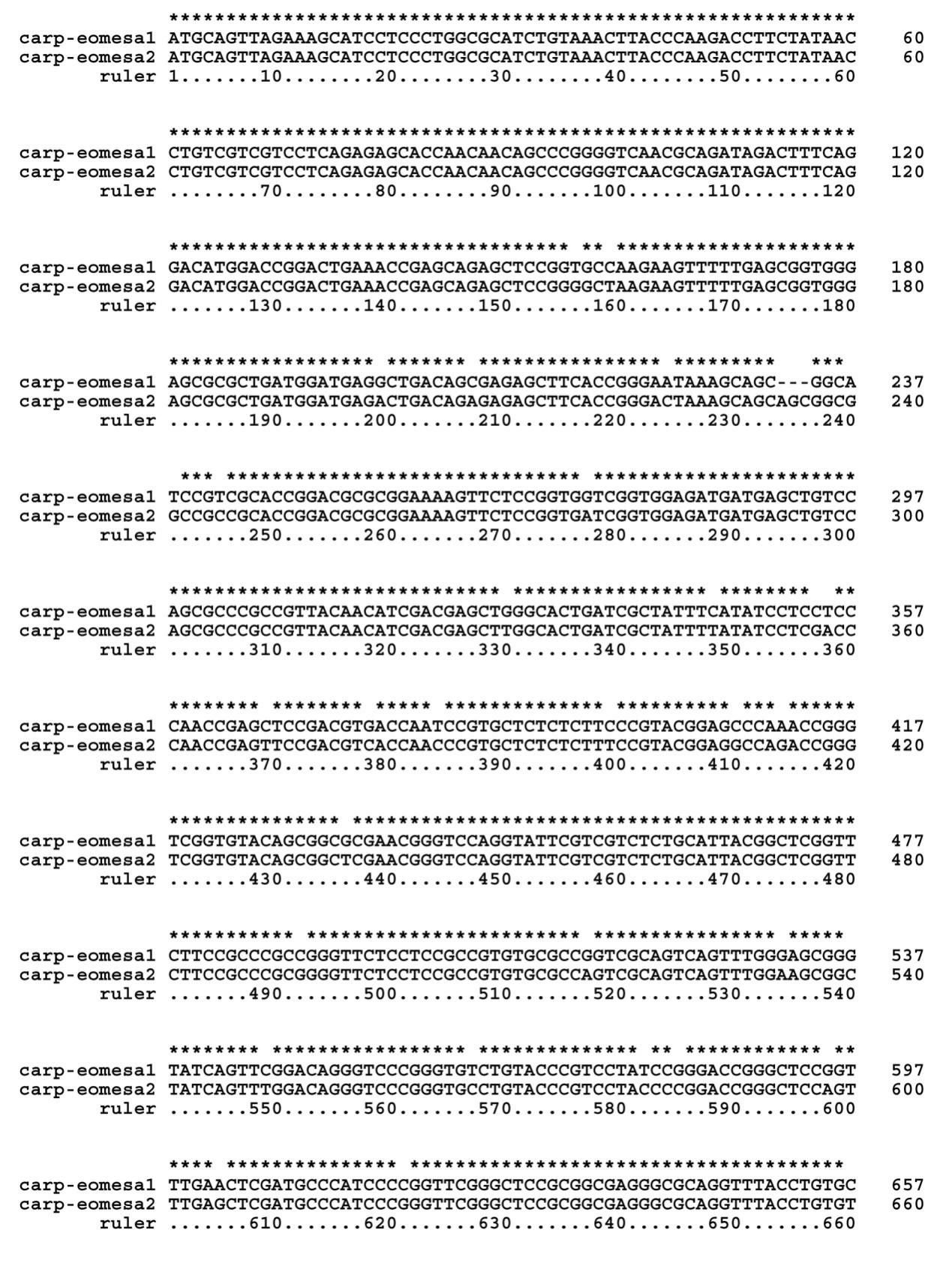


T1-F

T4-F

T1

T4-F

T2-F

T4

T1-R

T2


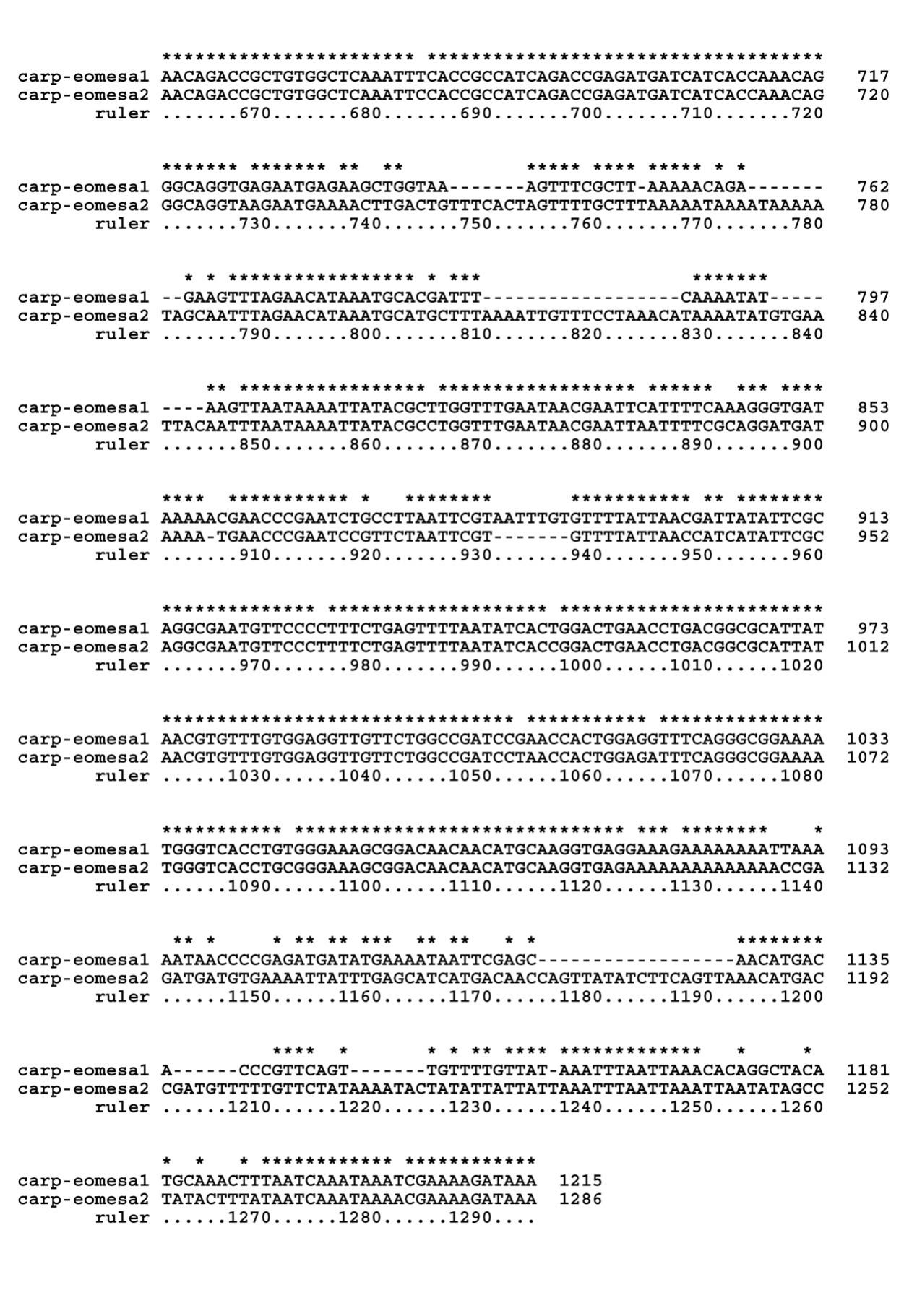


T3-R

T3-R

T3

T4-R/T2-R

T3-F

**S1 Fig. The position of four target sites and their locus-specific primer pairs.**
