# Supplementary material for: Disruption of T-box transcription factor eomesa results in abnormal development of median fins in Oujiang color common carp Cyprinus carpio
Source: PLoS One. 2023 Mar 2;18(3):e0281297. doi: 10.1371/journal.pone.0281297 (PMC9980737; doi:10.1371/journal.pone.0281297)
Supplement: S2 Fig — (DOCX) [file pone.0281297.s002.docx]

**The display of CCTop homepage**


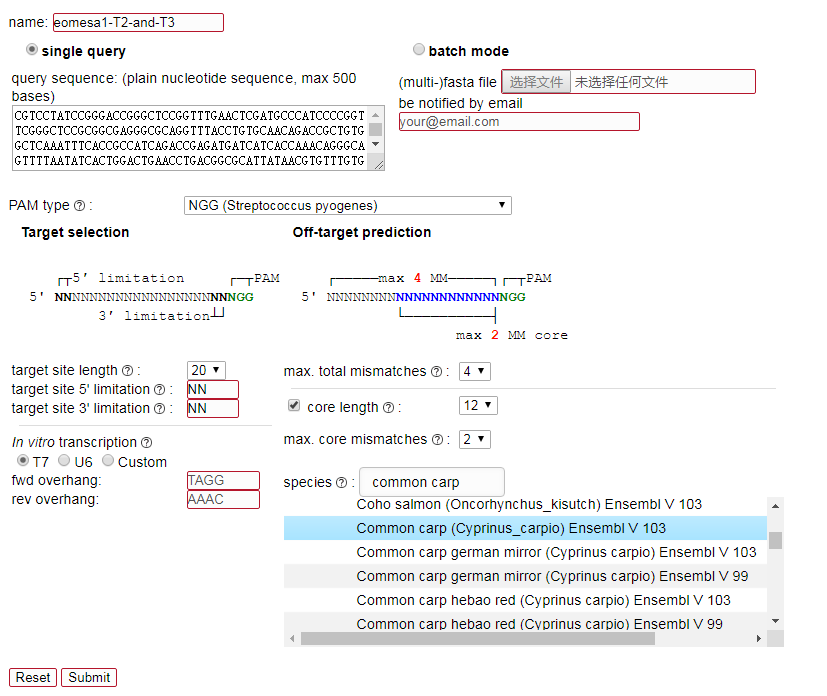


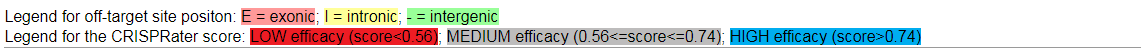


**T1 target and off-target information**


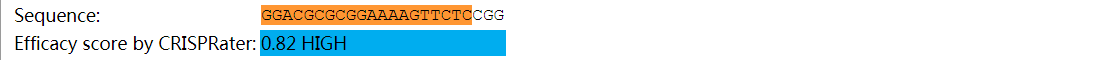


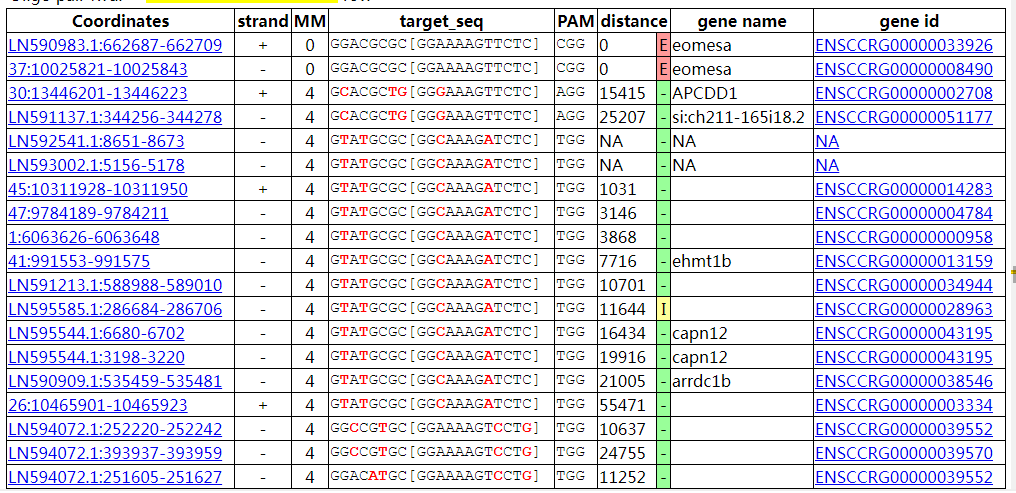


**T2 target and off-target information**


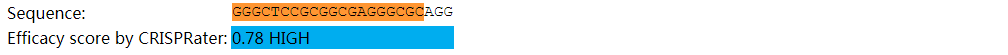


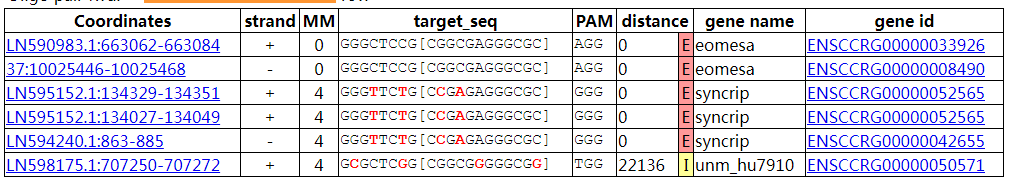


**T3 target and off-target information**


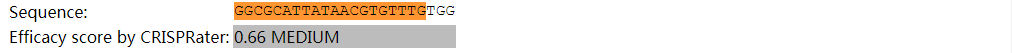


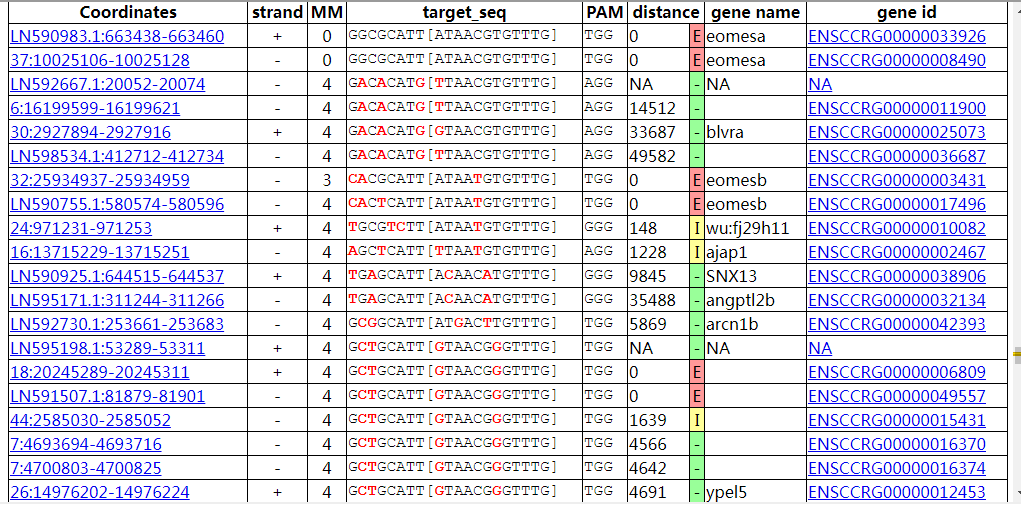


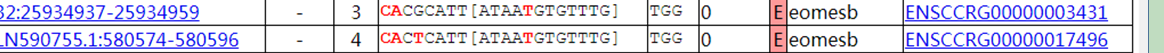


**T4 target and off-target information**


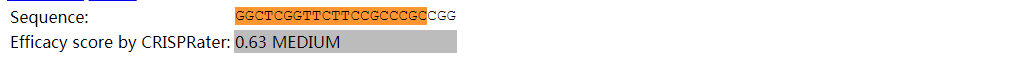


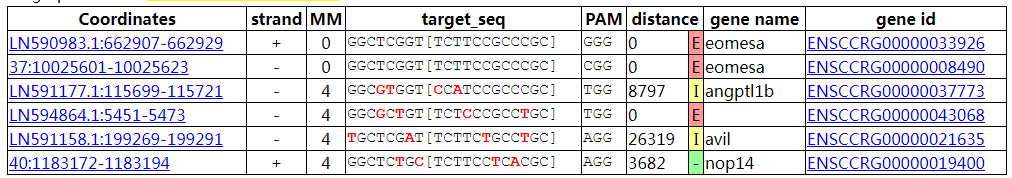


**S2 Fig. The information of four targets and their off-target sites presented in CCTop.**
