# Supplementary material for: Disruption of T-box transcription factor eomesa results in abnormal development of median fins in Oujiang color common carp Cyprinus carpio
Source: PLoS One. 2023 Mar 2;18(3):e0281297. doi: 10.1371/journal.pone.0281297 (PMC9980737; doi:10.1371/journal.pone.0281297)
Supplement: S3 Fig — (DOCX) [file pone.0281297.s003.docx]

**A T1 site**

**Eomesa1-T1 (24 hpf)**

**Reference:** CGGGAATAAAGCAGCGGCATCCGTCGCACCGGACGCGCGGAAAAGTTCTCCGGTGGTCGGTGGAGATGATGAGCTGTCCA

A1-T1-1-07: CGGGAATAAAGCAGCGGCATCCGTCGCACCGGACGCGCGG--------------------TGGAGATGATGAGCTGTCCA (-20 bp)

A1-T1-2-24: CGGGAATAAAGCAGCGGCATCCGTCGCACCGGACGCGCGGACGCGCGGAAAAGGTGGTCGGTGGAGATGATGAGCTGTC (+1 bp)

A1-T1-2-50: CGGGAATAAAGCAGCGGCATCCGTCGCACCGGACGCGCGGAAAA--------GTGGTCGGTGGAGATGATGAGCTGTCCA (-8 bp)

A1-T1-2-80: CGGGAATAAAGCAGCGGCATCCGTCGCACCGGACGCGCGGAAAAGTTACCCAAGACCGGTGGTCGGTGGAGATGATGAG (+6 bp)

A1-T1-2-82: CGGGAGTAAAGCAGCGGCATCCGTCGCACCGGACGCGCGGAAAAGTCGGTGGAGATGGTCGGTGGTCGGTGGAGATGAT (+9 bp)

A1-T1-2-83: CGGGAATAAAGCAGCGGCATCCGTCGCACCGGACGCGCGGAAAAGT-----GGTGGTCGGTGGAGATGATGAGCTGTCCA (-5 bp)

A1-T1-3-45: CGGGAATAAAGCAGCGGCATCCGTCGCACCGGACGCG-------------CGGTGGTCGGTGGAGATGATGAGCTGTTCAG (-13 bp)

A1-T1-3-68: CGGGAATAAAGCAGCGGCATCCGTCGCACCGGACGCGCGGAAAAGT---CCGGTGGTCGGTGGAGATGATGAGCTGTCCAG (-3 bp)

A1-T1-3-69: CGGGAATAAAGCAGCGGCATCCGTCGCACCGGACGCGCGGAAAAGTGGTGGCCGGTGGTCGGTGGAGATGATGAGCTGTCC (+2 bp)

A1-T1-3-71: CGGGAATAAAGCAGCGGCATCCGTCGCACCGGACGCGCGGAAAAGT---CCGGTGGTCGGTGGAGATGATGAGCTGTCCAG (-3 bp)

A1-T1-3-74: CGGGAATAAAGCAGCGGCATCCGTCGCACCGGACGCGCGGAAAAGTT----GGTGGTCGGTGGAGATGATGAGCTGTCCAG (-4 bp)

A1-T1-3-75: CGGGAATAAAGCAGCGGCATCCGTCGCACCGGACGCGCGGAAAAGT-----GGTGGTCGGTGGAGATGATGAGCTGTCCAG (-5 bp)

A1-T1-4-11: CGGGAATAAAGCAGCGGCATCCGTCGCACCGGACGCGCGGAAAAGTTCTCGTTTTAGAGCTAGAAATAGCAAGTTAAAATA

AGGCTAGTCCGTTATCAACTTGAAAAAGTGGCACCGGTGGTCGGTGGAGATGATGAGCTGTCCAGCGCCCGCCGTTACAACA (+65 bp)

A1-T1-4-12: CGGGAATAAAGCAGCGGCATCCGTCGCACCGGACGCGTGG-------TCCACGTGGTCGGTGGAGATGATGAGCTGTCCAGC (-7 bp)

A1-T1-4-55: CGGGAATAAAGCAGCGGCATCCGTCGCACCGGACGCGCGGAAAAGTT-------GGTCGGTGGAGATGATGAGCTGTCCAGC (-7 bp)

A1-T1-4-56: CGGGAATAAAGCAGCGGCATCCGTCGCACCGGACGCGCGGAAAAGTT-------GGTCGGTGGAGATGATGAGCTGTCCAGC (-7 bp)

A1-T1-4-57: CGGGAATAAAGCAGCGGCATCCGTCGCACCGGACGCGCGGAAAAGTTGCTTTCTAACTCCGGTGGTCGGTGGAGATGATGAG (+9 bp)

A1-T1-4-72: CGGGAATAAAGCAGCGGCATCCGTCGCACCGGACGCGCGGAAAAGTT-------GGTCGGTGGAGATGGTGAGCTGTCCAGC (-7 bp)

A1-T1-4-74: CGGGAATAAAGCAGCGGCATCCGTCGCACCGGACGCGCGGAAAAGTTC--CGGTGGTCGGTGGAGATGATGAGCTGTCCAGC (-2 bp)

A1-T1-4-76: CGGGAATAAAGCAGCGGCATCCGTCGCACCGGACGCGCGGAAGAGTTGGAAAACTCCGGTGGTCGGTGGAGATGATGAGCT (+6 bp)

A1-T1-4-77: CGGGAATAAAGCAGCGGCATCCGTCGCACCGGACGCGCGGAAAAGTTCATCAGTGGTCGGTGGTCGGTGGAGATGATGAGCT (+7 bp)

A1-T1-4-91: CGGGAATAAAGCAGCGGCATCCGTCGCACCGGACGCGCGGAAAAGT--------GGTCGGTGGAGATGATGAGCTGTCCAGC (-8 bp)

A1-T1-5-13: CGGGAATAAAGCAGCGGCATCCGTCGCACCGGACGCGCGGAAAAGT---CCGGTGGTCGGTGGAGATGACGAGCTGTCCAGC (-3 bp)

A1-T1-5-14: CGGGAATAAAGCAGCGGCATCCGTCGCACCGGACGCGCGGAAAAGTTGTCTGTGTCTTGTGCACTGTTTCTCCGGTGGTCGGT (+22 bp)

A1-T1-5-15: CGGGAATAAAGCAGCGGCATCCGTCGCACCGGACGCGCGGACG----CTCCGGTGGTCGGTGGAGATGATGAGCTGTCCAGC (-4 bp)

A1-T1-5-52: CGGGAATAAAGCAGCGGCATCCGTCGCACCGGACGCGCGGAAAAGTTTT---GTGGTCGGTGGAGATGATGAGCTGTCCAGCG (-3 bp)

A1-T1-5-53: CGGGAATAAAGCAGCGGCATCCGTCGCGCCGGACGCGCGGAAAAGTTC--CGGTGGTCGGTGGAGATGATGAGCTGTCCAGC (-2 bp)

A1-T1-5-54: CGGGAATAAAGCAGCGGCATCCGTCGCACCGGACGCGCGGAAAAGTGGT-CGGTGGTCGGTGGAGATGATGAGCAGTCCAGC (-1 bp)

A1-T1-5-55: CGGGAATAAAGCAGCGGC--------------------------CTCCGGTGGTCGGTGGAGATGATGAGCTGTCCAGCGCCC (-26 bp)

A1-T1-5-78: CGGGAATAAAGCAGCGGCATCCGTCGCACCGGACGCGCGGAAAAGTTTCATAGAACTGATGGTCGGTGGTCGGTGGAGATGAT (+13 bp)

A1-T1-5-79: CGGGAATAAAGCAGCGGCATCCGTCGCACCGGAC--------------TCCGGTGGTCGGTGGAGATGATGAGCTGTCCAGCC (-14 bp)

A1-T1-5-80: CGGGAATAAAGCAGCGGCATCCGTCGCACCGGACGCGCGGAAAAGTTTCATAGAACTGATGGTCGGTGGTCGGTGGAGATGAT (+13 bp)

A1-T1-5-81: CGGGAATAAAGCAGCGGCATCCGTCGCACCGGACGCG-------------CGGTGGTCGGTGGAGATGATGAGCTGTCCAGC (-13 bp)

A1-T1-5-83: CGGGAATAAAGCAGCGGCACCCGTCGCACCGGACGCGCAGAAAAGTGGT-CGGTGGTCGTTGGAGACGATGAGCTGTCCAGC (-1 bp)

A1-T1-5-90: CGGGAATAAAGCAGCGGCATCCGTCGCACCGGACGCGCGGAAAAGCCACAGCGACTGACTGTTTTCTCCGGTGGTCGGTGGA (+18 bp)

**Eomesa1-T1 (7 dpf)**

**Reference:** CGGGAATAAAGCAGCGGCATCCGTCGCACCGGACGCGCGGAAAAGTTCTCCGGTGGTCGGTGGAGATGATGAGCTGTCCA

A1-T1-5-07: CGGGAATAAAGCAGCGGCATCCGTCGCACCGGACGCGCGGAAAAGTTGCTCCGGTGGTCGGTGGAGATGATGAGCTGTCC (+1 bp)

A1-T1-5-05: CGGGAATAAAGCAGCGGCATCCGTCGCACCGGACGCGCGGAAAAGT---CCGGTGGTCGGTGGAGATGATGAGCTGTCCA (-3 bp)

A1-T1-5-11: CGGGAATAAAGCAGCGGCATCCGTCGCACCGGACGCGCGGAAAAGTT-----GTGGTCGGTGGAGATGATGAGCTGTCCA (-5 bp)

A1-T1-5-08: CGGGAATAAAGCAGCGGCATCCGtcgcaccggacgcgcggaaaagt--------ggtcggtggagatgatgagctgtcca (-8 bp)

A1-T1-5-01: CGGGAATAAAGCAGCGGCATCCGtcgcaccggacgcca---------ctccggtggtcggtggagatgatgagctgtcca (-9 bp)

A1-T1-5-09: CGGGAATAAAGCAGCGGCATCCGtcgcaccggac-cac----------ctccggtggtcggtggagatgatgagctgtcca (-10 bp)

A1-T1-5-06: CGGGAATAAAGCAGCGGCATCCGtcgcaccggacgcgcgg-------------tggtcggtggagatgatgagctgtcca (-13 bp)

A1-T1-5-04: CGGGAATAAAGCAGCGGCATCCGtcgcaccggacgcca---------ctccggtggtcggtggagatgatgagctgtcca (-9 bp)

A1-T1-6-06: CGGGAATAAAGCAGCGGCATCCGtcgcaccggacgcgcggaaaagtccgcgcggtggtcggtggagatgatgagctgtcc (+1 bp)

A1-T1-6-05: CGGGAATAAAGCAGCGGCATCCGtcgcaccggacgcgcggaaaagttggtccggtggtcggtggagatgatgagctgtcc (+1 bp)

A1-T1-6-03: CGGGAATAAAGCAGCGGCATCCGtcgcaccggacgcgcggaaaagtccgcgcggtggtcggtggagatgatgagctgtcc (+1 bp)

A1-T1-6-04: CGGGAATAAAGCAGCGGCATCCGtcgcaccggacgcgcggaaaagtt----ggtggtcggtggagatgatgagctgtcca (-4 bp)

A1-T1-6-01: CGGGAATAAAGCAGCGGCATCCGtcgcaccggacgcgcggaaaagttggtccggtggtcggtggagatgatgagctgtcca (+1 bp)

A1-T1-6-09: CGGGAATAAAGCAGCGGCATCCGtcgcaccggacgcgcggaaaagtt-----gtggtcggtggagatgatgagctgtccag (-5 bp)

A1-T1-6-10: CGGGAATAAAGCAGCGGCATCCGtcgcaccggacgcgcggaaaagt-----------cggtggagatgatgagctgtccag (-11 bp)

A1-T1-6-08: CGGGAATAAAGCAGCGGCATCCGTCGCACCGGACCGTTATCAACTTGAAAAAGTGGCACCGAGTCGGTGCTTTTTTCCGGTG

TCGGTGGTCGGTGGAGATGATGAGCTGTCCAGCGCCCGCCGTTACAACATCGACGAGCTGGGCACTGATCGCTATTTCATATC (+34bp) A1-T1-6-02: CGGGAATAAAGCAGCGGCATC-----------------------------CGGTGGTCGGTGGAGATGATGAGCTGTCCAGC (-29 bp)

A1-T1-6-07: CGGGAATAAAGCAGCGGCATCCGTCGCACCGGACGCGCGGAAAAGTT-----------------------------------

----------ACAACATCGACGAGCTGGGCACTGATCGCTATTTCATATCCTCCTCCCAACCGAGCTCCGACGTGACCAATCCG (-45 bp)

A1-T1-7-02: CGGGAATAAAGCAGCGGCATCCGtcgcaccggacgcgcggaaaagtttctccggtggtcggtggagatgatgagctgtcca (+1 bp)

A1-T1-7-11: CGGGAATAAAGCAGCGGCATCCGtcgcaccggacgcgcggaaaagttcatcatctccggtggtcggtggagatgatgagctg (+6 bp)

A1-T1-7-07: CGGGAATAAAGCAGCGGCATCCGtcgcaccggacgcgcggaaaag-------gtggtcggtggagatgatgagctgtccag (-7 bp)

A1-T1-7-09: CGGGAATAAAGCAGCGGCATCCGtcgcaccggacgcgc----------tccggtggtcggtggagatgatgagctgtccagc (-10 bp)

A1-T1-7-01: CGGGAATAAAGCAGCGGCATCCGtcgcaccggatgcgcggaaaa------ccatggtcggtggagatgatgagctgtccagc (-6 bp)

A1-T1-7-12: CGGGAATAAAGCAGCGGCATCCGtcgcaccggacgcgcggaactcggactccaccgaccatctccggtggtcggtggagat (+14 bp)

A1-T1-7-10: CGGGAATAAAGCAGCGGCATCCGtcgcaccg---------------------gtggtcggtggagatgatgagctgtccagc (-21 bp)

A1-T1-7-05: CGGGAATAAAGCAGCGGCATCCGtcgcaccg---------------------gtggtcggtggagatgatgagctgtccagc (-21 bp)

A1-T1-7-04: CGGGAATAAAGCAGCGGCATCCGtcgcaccg---------------------gtggtcggtggagatgatgagctgtccagc (-21 bp)

**Eomesa2-T1 (24 hpf)**

**Reference:**  GACTAAAGCAGCAGCGGCGGCCGCCGCACCGGACGCGCGGAAAAGTTCTCCGGTGATCGGTGGAGATGATGAGCTGTCCA

A2-T1-1-38: GACTAAAGCAGCAGCGGCGGCCGCCGCACCGGACGCGCGGAAAAGT---CCGGTGATCGGTGGAGATGATGAGCTGTCC (-3 bp)

A2-T1-1-41: GACTAAAGCAGCAGCGGCGGCCGCCGCACCGGACGTGCGGAAAAGTT---------GAGATGGAGATGATGAGCTGTCC (-9 bp)

A2-T1-1-58: GACTAAAGCAGCAGCGGCGGCCGCCGCACCGGACGCGCGGAAAAGT---CCGGTGATCGGTGGAGATGATGAGCTGTCC (-3 bp)

A2-T1-1-61: GACTAAAGCAGCAGCGGCGGCCGCCGCACCGGACGCGCGGAAAAGTTGTCCATCATCTCCGGTGATCGGTGGAGATGAT (+9 bp)

A2-T1-1-63: GACTAAAGCAGCAGCGGCGGCCGCCGCACCG---------------------GTGATCGGTGGAGATGATGAGCTGTCCA (-21 bp)

A2-T1-1-65: GACTAAAGCAGCAGCGGCGGCCGCCGCACCGGACGCGCGGAAAAGTA----GGTGATCGGTGGAGATGATGAGCTGTCC (-4 bp)

A2-T1-1-66: GACTAAAGCAGCAGCGGCGGCCGCCGCACCGGACGCGCGGAAAAGTTGATCCGGTGATCGGTGGAGATGATGAGCTGTC (+1 bp)

A2-T1-1-67: GACTAAAGCAGCAGCGGCGGCCGCCGCACCGGACGCGCGGAAAAG-------GTGATCGGTGGAGATGATGAGCTGTCC (-7 bp)

A2-T1-2-34: GACTAAAGCAGCAGCGGCGGCCGCCGCACCGGACGCGCGGAAAAGTTTT --GATGTTCGGTGGAGATGATGAGCTGTCC (-2 bp)

A2-T1-2-36: GACTAAAGCAGCAGCGGCGGCCGCCGCACCGGACGCGCTCAAAAA--CTCCGGTGATCGGTGGAGATGATGAGCTGTCC (-2 bp)

A2-T1-2-37: GACTAAAGCAGCAGCGGCGGCCGCCGCACCG---------------------GTGATCGGTGGAGATGATGAGCTGTCCA (-21 bp)

A2-T1-2-48: GACTAAAGCAGCAGCGGCGGCCGCCGCACCGGACGCGCGGAAAAGATGTTTCTCCGGTGATCGGTGGAGATGATGAGCT (+4 bp)

A2-T1-2-50: GACTAAAGCAGCAGCGGCGGCCGCCGCACCGGACGCGCGGAAAAGTTGGAGAGGTGATCGGTGGAGATGATGAGCTGTC (+1 bp)s

A2-T1-2-51: GACTAAAGCAGCAGCGGCGGCCGCCGCACCGGACGCGCGGAAAA------------TCGGTGGAGATGATGAGCTATCC (-12 bp)

A2-T1-2-52: GACTAAAGCAGCAGCGGCGGCCGCCGCACCGGACGCGCGGAAAAGT--------GATCGGTGGAGATGATGAGCTGTCC (-8 bp)

A2-T1-2-54: GACTAAAGCAGCAGCGGCGGCCGCCGCACCGGACGCGCGGAAAAGTT-------GATCGGTGGAGATGATGAGCTGTCC (-7 bp)

A2-T1-2-55: GACTAAAGCAGCAGCGGCGGCCGCCGCACCGGACGCGCGGAAAAGT--------GATCGGTGGAGATGATGAGCTGTCC (-8 bp)

A2-T1-2-56: GACTAAAGCAGCAGCGGCGGCCGCCGCACCGGACGCGCGGA-------------GATCGGTGGAGATGATGAGCTGTCC (-13 bp)

A2-T1-2-57: GACTAAAGCAGCAGCGGCGGCCGCCGCACCGGACGCGCGGAAAAGTTTTTTCCGGTGATCGGTGGAGATGATGAGCTGT (+2 bp)

A2-T1-3-31: GACTAAAGCAGCAGCGGCGGCCGCCGCACCGGACGC------------TCCGGTGATCGGTGGAGATGATGAGCTGTCC (-12 bp)

A2-T1-3-44: GACTAAAGCAGCAGCGGCGGCCGCCGCACCGGACGCGCGGAAAAGTTCATTGATCTCCGGTGATCGGTGGAGATGATGA (+7 bp)

A2-T1-4-14: GACTAAAGCAGCAGCGGCGGCCGCCGCACCGGACGCGCGGAAAAGCGGTGATCGGTGATCGGTGGAGATGATGAGCTG (+2 bp)

A2-T1-4-49: GACTAAAGCAGCAGCGGCGGCCGCCGCACCGGACGCGCGGAAAAGT--------GATCGGTGGAGATGATGAGCTGTCC (-8 bp)

A2-T1-4-71: GACTAAAGCAGCAGCGGCGGCCGCCGCACCGGACGCGCGGAA--------------TCGGTGGAGATGATGAGCTGTCC (-14 bp)

A2-T1-4-72: GACTAAAGCAGCAGCGGCGGCCGCCGCACCGGACGCGCGGAAAAGT---CCGGTGATCGGTGGAGATGATGAGCTGTCC (-3 bp)

A2-T1-4-73: GACTAAAGCAGCAGCGGCGGCCGCCGCACCGGACGGCCGCCGCA-----CTCCGGTGATCGGTGGAGATGATGAGCTGT (-5,+2 bp)

A2-T1-4-79: GACTAAAGCAGCAGCGGCGGCCGCCGCACCGGACGCGCGGAAAAGTT-----GTGATCGGTGGAGATGATGAGCTGTCC (-5 bp)

A2-T1-5-07: GACTAAAGCAGCAGCGGCGGCCGCCGCACCGGACGCGCGGACA------CCGGTGATCGGTGGAGATGATGAGCTGTCC (-6 bp)

A2-T1-5-08: GACTAAAGCAGCAGCGGCGGCCGCCGCACCGGACGCGC-------TCCACCGGTGATCGGTGGAGATGATGAGCTGTCC (-7 bp)

A2-T1-5-19: GACTAAAGCAGCAGCGGCGGCCGCCGCACCGGACGCGCGGACGCGCGGACGACTCGGTGCCACTCCGGTGATCGGTGG (+15 bp)

A2-T1-5-73: GACTAAAGCAGCAGCGGCGGCCGCCGCACCGGACGCGCGGAAAAGT--------GATCGGTGGAGATGATGAGCTGTC (-8 bp)

A2-T1-5-74: GACTAAAGCAGCAGCGGCGGCCGCCGCACCGGACGCGCGGAAAAGAGAGAGCACGTGTTCTCCGGTGATCGGTGGAGA (+12 bp)

A2-T1-5-76: GACTAAAGCAGCAGCGGCGGCCGCCGCACCGGACGCGCGGAAAAG------------GTGGAGATGATGAGCTGTCCA (-14 bp)

A2-T1-5-78: GACTAAAGCAGCAGCGGCGGCCGCCGCACCGGACGCGCGGAAAAGTTCATCATCTCCGGTGATCGGTGGAGATGATGA (+6 bp)

A2-T1-5-79: GACTAAAGCAGCAGCGGCGGCCGCCGCACCGGACGCGCGG--------------------TGGAGATGATGAGCTGTCC (-20 bp)

A2-T1-5-80: GACTAAAGCAGCAGCGGCGGCCGCCGCACCGGACGCGCGG--------------------TGGAGATGATGAGCTGTCC (-20 bp)

A2-T1-5-82: GACTAAAGCAGCAGCGGCGGCCGCCGCACCGGACGCGCGGAAAAGT--------GATCGGTGGAGATGATGAGCTGTCC (-8 bp)

A2-T1-6-03: GACTAAAGCAGCAGCGGCGGCCGCCGCACCGGACGCGCGGAAAAGTTTTCCATCTCCATCTCCGGTGATCGGTGGAGAT (+12 bp)

**Eomesa2-T1 (7 dpf)**

**Reference:** GACTAAAGCAGCAGCGGCGGCCGCCGCACCGGACGCGCGGAAAAGTTCTCCGGTGATCGGTGGAGATGATGAGCTGTCCA

A2-T1-5-08: GACTAAAGCAGCAGCGGCGGCcgccgcaccggacgcgcggaaaagttcaccgatcggtgatcggtggagatgatgagctg (+4 bp)

A2-T1-5-06: GACTAAAGCAGCAGCGGCGGCcgccgcaccggacgcgcggaaaagtgatc-ggtgatcggtggagacgatgagctgtcca (-1 bp)

A2-T1-5-03: GACTAAAGCAGCAGCGGCGGCcgccgcaccggacgcgcggaaaagagagagccggtgatcggtggagatgatgagctgtc (+2 bp)

A2-T1-5-07: GACTAAAGCAGCAGCGGCGGCcgccgcaccggacgcgcggaaaagt---ccggtgatcggtggagatgatgagctgtcca (-3 bp)

A2-T1-5-09: GACTAAAGCAGCAGCGGCGGCcgccgcaccggacgcgcggaaaagtt-------gatcggtggagatgatgagctgtcca (-7 bp)

A2-T1-5-05: GACTAAAGCAGCAGCGGCGGCcgccgcaccggacgcgcggagat------cggtgatcggtggagatgatgagctgtcca (-6 bp)

A2-T1-5-11: GACTAAAGCAGCAGCGGCGGCcgccgcaccggacgc-------------ccggtgatcggtggagatgatgagctgtcca (-13 bp)

A2-T1-5-01: GACTAAAGCAGCAGCGGCGGCcgccgcaccggacgc-------------ccggtgatcggtggagatgatgagctgtcca (-13 bp)

A2-T1-6-09: GACTAAAGCAGCAGCGGCGGCcgccgcaccgt-cgcgcggaaaagttctccggtgatcggtggagatgatgagctgtcca (-1 bp)

A2-T1-6-10: GACTAAAGCAGCAGCGGCGGCcgccgcaccggacgcgcggaaaagttc---ggtgatcggtggagatgatgagctgtcca (-3 bp)

A2-T1-6-11: GACTAAAGCAGCAGCGGCGGCcgccgcaccggacgcgcggaaaagttccaccgatctcctccggtgatcggtggagatga (+11 bp)

A2-T1-6-04: GACTAAAGCAGCAGCGGCGGCcgccgcaccggacgcgcggaaaagt--------gatcggtggagatgatgagctgtcca (-8 bp)

A2-T1-6-12: GACTAAAGCAGCAGCGGCGGCcgccgcaccggacgcgcggaaaagttccaccgatctcctccggtgatcggtggagatga (+11 bp)

A2-T1-6-06: GACTAAAGCAGCAGCGGCGGCcgccgcaccggacgcgcggaaatcggtgatcggtgatcggtggagatgatgagctgtcc (+1 bp)

A2-T1-6-05: GACTAAAGCAGCAGCGGCGGCcgccgcaccggacgcgcggaaatcggtgatcggtgatcggtggagatgatgagctgtcc (+1 bp)

A2-T1-6-07: GACTAAAGCAGCAGCGGCGGCcgccgcaccggacgcgcgg-------------tgatcggtggagatgatgagctgtcca (-13 bp)

A2-T1-7-11: GACTAAAGCAGCAGCGGCGGCcgccgcaccggacgcgcggaaaagttcatgtccggtgatcggtggagatgatgagctgt (+3 bp)

A2-T1-7-04: GACTAAAGCAGCAGCGGCGGCcgccgcaccggacgcgcggaaaagtt----ggtgatcggtggagatgatgagctgtcca (-4 bp)

A2-T1-7-05: GACTAAAGCAGCAGCGGCGGCcgccgcaccggacgcgcggaaaagtt-------gatcggtggagatgatgagctgtcca (-7 bp)

A2-T1-7-02: GACTAAAGCAGCAGCGGCGGCcgccgcaccggacgcgcggaaaagtt-------gatcggtggagatgatgagctgtcca (-7 bp)

A2-T1-7-07: GACTAAAGCAGCAGCGGCGGCcgccgcaccggacgcgcggaaaagt--------gatcggtggagacgatgagctgtcca (-8 bp)

A2-T1-7-06: GACTAAAGCAGCAGCGGCGGCcgccgcaccggacgcgcggaaaagtt-------gatcggtggagatgatgagctgtcca (-7 bp)

A2-T1-7-12: GACTAAAGCAGCAGCGGCGGCcgccgcaccggacgcgcggagatgatt-------atcggtggagatgatgagctgtccag (-7 bp)

A2-T1-7-08: GACTAAAGCAGCAGCGGCGGCcgccgcaccggacgcgcgg-------------tgatcggtggagatgatgagctgtccag (-13 bp)

A2-T1-7-10: ATGAGACTGACA---------------------------------------------------------------CTCCGGTG

ATCGGTGGAGATGATGAGCTGTCCAGCGcccgccgttacaacatcgacgagcttggcactgatcgctattttatatcctcgacc (-63 bp)

**B T2 site**

**Eomesal-T2 (24 hpf)**

**Reference:** CGGTTTGAACTCGATGCCCATCCCCGGTTCGGGCTCCGCGGCGAGGGCGCAGGTTTACCTGTGCAACAGACCGCTGTGGC

A1-T2-1-21: CGGTTTGAACTCGATGCCCATccccggttcgggctccgcggcg-------aggtttacctgtgcaacagaccgctgtggcT (-7 bp)

A1-T2-1-32: CGGTTTGAACTCGATGCCCATccccggttcgggctccgc------ggcgcaggtttacctgtgcaacagaccgctgtggct (-6 bp)

A1-T2-1-33: CGGTTTGAACTCGATGCCCATccccggttcgggctccgc------ggcgcaggtttacctgtgcaacagaccgctgtggct (-6 bp)

A1-T2-1-37: CGGTTTGAACTCGATGCCCATccccggttcgggctccgcggcg-------aggtttacctgtgcaacagaccgctgtggcT (-7 bp)

A1-T2-2-20: CGGTTTGAACTCGATGCCCATCCCCGGTTCGGGCTCCGCGGCGAGGGAAAAGTTCTCCGGTGGTCGGTGGCAGGTTTACC (+21 bp)

A1-T2-2-27: CGGTTTGAACTCGATGCCCATCCCCGGTTCGGGCTCCGCGGGATGGGCAAAGTTCTCCGGTGGTCGGTG----ATCGAGT (-4 bp)

A1-T2-3-10: CGGTTTGAGCTCGATGCCCATCCCCGGTTCGGGCTCCGCGGCGAGGGTTTACCTGTGCAACAGACCGCTGTGCAACAGAC

CGCTGAGGTTTACCTGTGCAACAGACCGCTGTGGCTCAAATTTCACCGCCATCAGACCGAGATGATCATCACCAAACAGGG (+35 bp)

A1-T2-3-11: CGGTTTGAACTCGATGCCCATCCCCGGTTCGGGCTCCGCGGCGAGGATCCGCAGGTTTACCTGTGCAACAGACCGCTGTG (+2 bp)

A1-T2-3-12: CGGTTTGAACTCGATGCCCAtccccggttcgggctccgcggcgagttttaccgcggcaggtttacctgtgcaacagaccg (+7 bp)

A1-T2-3-14: CGGTTTGAACTCGATGCCCAtccccggttcgggctccgcggcgacagagtgcaacagctacctgtgggctacctgtgcaa (+14 bp)

A1-T2-3-15: CGGTTTGAACTCGATGCCCAtccccggttcgggctccgcggcgagg-----ggtttacctgtgcaacagaccgctgtggc (-5 bp)

A1-T2-3-16: CGGTTTGAACTCGATGCCCATccccggttcgggctccgcggcgatgttgcgcaggtttacctgtgcaacagaccgctgtgg (+2 bp)

A1-T2-3-18: CGGTTTGAACTCGATGCCCAtccccggttcgggctccgcggcgatgttgcgcaggtttacctgtgcaacagaccgctgtgg (+2 bp)

A1-T2-4-48: CGGTTTGAACTCGATGCCCATCCCCGGTTCGGGCTCCGCGGCGAGG-----GGTTTACCTGTGCAACAGACCGCTGTGGCT (-5 bp)

A1-T2-4-45: CGGTTTGAACTCGATGCCCATCCCCGGTTCGGGCTCCGCGGCGAG------GGTTTACCTGTGCAACAGACCGCTGTGGCT (-6 bp)

A1-T2-4-26: CGGTTTGAACTCGATGCCCATCCCCGGTTCGGGCTC-----------CGCAGGTTTACCTGTGCAACAGACCGCTGTGGCT (-11 bp)

A1-T2-4-16: CGGTTTGAACTCGATGCCCATCCCCGGTTCGGGCTCCGCGGC----------TGTTACCTGTGCAACAGACCGCTGTGGCT (-10 bp)

A1-T2-4-23: CGGTTTGAACTCGATGCCCATCCCCGGTTCGGGTTCCGC------GGCGCAGGTTTACCTGTGCAACAGACCGCTGTGGCT (-6 bp)

A1-T2-4-17: CGGTTTGAACTCGATGCCCATCCCCGGTTCGGGCTCCGCGGCGAGGG-----------------AACAGACCGCTGTGGCT (-17 bp) A1-T2-4-22: CGGTTTGAACTCGATGCCCATCCCCGGTTCGGGCTCCGCGCCCTCGCCGCGGAGCCCTATAGTGAGTCTGTAAAAACGGAG

CCCTATAGTGAGTCTGTGTTTACCTGTAGCCCTATAGTTTACCTGTGCAACAGACCGCTGTGGCTCAAATTTCACCGCCATCA (+65 bp)

A1-T2-5-20: CGGTTTGAACTCGATGCCCATCCCCGGTTCGGGCTCCGC------GGCGCAGGTTTACCTGTGCAACAGACCGCTGTGGCT (-6 bp)

A1-T2-5-32: CGGTTTGAACTCGATGCCCATCCCCGGTTCGGGCTCCGCGGCGAG---GAAGGTTTACCTGTGCAACAGACCGCTGTGGCT (-3 bp)

A1-T2-6-52: CGGTTTGAACTCGATGCCCATCCCCGGTTCGGGCTCCGCGGCGAGGAGCCCGCAGGTTTACCTGTGCAACAGACCGCTGTG (+3 bp)

A1-T2-6-34: CGGTTTGAACTCGATGCCCATCCCCGGTTCGGGCTCCGCGGCGA-GGCG-AGGTTTACCTGTGCAACAGACCGCTGTGGCt (-2 bp)

A1-T2-6-51: CGGTTTGAACTCGATGCCCATCCCCGGTTCGGGCTCCGCGGCGAG------GGTTTACCTGTGCAACAGACCGCTGTGGCT (-6 bp)

A1-T2-6-33: CGGTTTGAACTCGATGCCCATCCCCGGTTCGGGCTCCGCGGCGAG------GGTTTACCTGTGCAACAGACCGCTGTGGCT (-6 bp)

A1-T2-6-34: CGGTTTGAACTCGATGCCCATCCCCGGTTCGGGCTCC---------GCGCAGGTTTACCTGTGCAACAGACCGCTGTGGCTc (-9 bp)

A1-T2-6-50: CGGTTTGAACTCGATGCCCATCCCCGGTTCGGGCTCCGC-----TGTTGCAGGTTTACCTGTGCAACAGACCGCTGTGGCTc (-5 bp)

A1-T2-6-37: CGGTTTGAACTCGATGCCCATCCCCGGTTCGGGCTCCGCGGCGAGGGAGCTCCCAAACCTGTTGCACAGGTTTACCTGTGC (+17 bp)

A1-T2-6-35: CGGTTTGAGCTCGATGCCCATCCCCGGTTCGGGC------------------------------ACAGACCGCTGTGGCTca (-30 bp)

**Eomesal-T2 (7 dpf)**

**Reference:** CGGTTTGAACTCGATGCCCATCCCCGGTTCGGGCTCCGCGGCGAGGGCGCAGGTTTACCTGTGCAACAGACCGCTGTGGC

A1-T2-1-02: CGGTTTGAACTCGATGCCCAtccccggttcgggctccgcggcgag---gcaggtttacctgtgcaacagaccgctgtggc (-3 bp)

A1-T2-1-08: CGGTTTGAACTCGATGCCCAtccccggttcgggctccgcggcgagggagcccaggtttacctgtgcaacagaccgctgtg (+2 bp)

A1-T2-1-04: CGGTTTGAACTCGATGCCCAtccccggttcgggctccgcggcgaggg-------ttacctgtgcaacagaccgctgtggc (-7 bp)

A1-T2-1-10: CGGTTTGAACTCGATGCCCAtccccggttcgggctccgcggcgagggtttacctgtgcaacaggtttacctgtgcaacag (+11 bp)

A1-T2-1-05: CGGTTTGAACTCGATGCCCAtccccggttcgggctccgcggcgaggtttacctgtgcaacaggtttacctgtgcaacaga (+10 bp)

A1-T2-1-11: CGGTTTGAACTCGATGCCCAtccccggttcgggctccgcggcgagggaggtaaacgcggcgcaggtttacctgtgcaaca (+12 bp)

A1-T2-1-01: CGGTTTGAACTCGATGCCCAtccccggttcgggctccgcggcgagggcggacgcaaacgacgacgcaggtttacctgtgc (+16 bp)

A1-T2-1-12: CGGTTTGAACTCGATGCCCAtccccggttcgggctccgcg--------gcaggtttacctgtgcaacagaccgctgtggc (-8 bp)

A1-T2-3-02: CGGTTTGAACTCGATGCCCAtccccggttcgggctccgcggcg-------aggtttacctgtgcaacagaccgctgtggc (-7 bp)

A1-T2-3-07: CGGTTTGAACTCGATGCCCAtccccggttcgggctc-----------cgcaggtttacctgtgcaacagaccgctgtggc (-11 bp)

A1-T2-3-05: CGGTTTGAACTCGATGCCCAtccccggttcgggctccgcggcga------------------gcaacagaccgctgtggc (-18 bp)

A1-T2-3-04: CGGTTTGAACTCGATGCCCAtccccggttcgggctccgcggcgagggtccgtggcaggtttacctgtgcaacagaccgct (+5 bp)

A1-T2-3-03: CGgtttgaactcgatgcccatccccggttc---------------------------------------------------

--------accgccaTcagaccgagatgatcatcaccaaacagggcaggtgagaatgagaagctggtaaagtttcgctta (-59 bp)

A1-T2-4-10: CGGTTTGAACTCGATGCCCAtccccggttcgggctccgcggcgagggttacctgcgcaggtttacctgtgcaacagaccg (+7 bp)

A1-T2-4-08: CGGTTTGAACTCGATGCCCAtccccggttcgggctccgc------ggcgcaggtttacctgtgcaacagaccgctgtggc (-6 bp)

A1-T2-4-06: CGGTTTGAACTCGATGCCCAtccccggttcgggctccgc------ggcgcaggtttacctgtgcaacagaccgctgtggc (-6 bp)

A1-T2-4-02: CGGTTTGAACTCGATGCCCAtccccggttcgggctccgcggcgag------ggtttacctgtgcaacagaccgctgtggc (-6 bp)

A1-T2-4-01: CGGTTTGAACTCGATGCCCAtccccggttcgggctccgc------ggcgcaggtttacctgtgcaacagaccgctgtggc (-6 bp)

A1-T2-4-12: CGGTTTGAACTCGATGCCCAtccccggttcgggctccgcggcgag------ggtttacctgtgcaacagaccgctgtggct (-6 bp)

A1-T2-4-13: CGGTTTGAACTCGATGCCCAtccccggttcgggctccgcggcgagtccgttatcattatcaggtttacctgtgcaacaga (+10 bp)

A1-T2-4-15: CGGTTTGAACTCGATGCCCAtccccggttcgggctccgc------------ggtttacctgtgcaacagaccgctgtggc (-12 bp)

A1-T2-4-05: CGGTTTGAACTCGATGCCCAtccccggttcgggctccgcg----ggctccaggt-------------agaccgctgtggc (-17 bp)

**Eomesa2-T2 (24 hpf)**

**Reference**: CAGTTTGAGCTCGATGCCCATCCCGGGTTCGGGCTCCGCGGCGAGGGCGCAGGTATACCTGTGTAACAGACCGCTGTGGC

A2-T2-1-01: CAGTTTGAGCTCGATGCCCATCCCGGGTTCGGGCTCCACGGCGAGGGTTACACAGGGTTTACCTGTGTAACAGACCGCTG (+4 bp)

A2-T2-1-09: CAGTTTGAGCTCGATGCCCATCCCGGGTTCGGGCTCCGCGGCGAGGG------TTTACCTGTGTAACAGACCGCTGTGGC (-6 bp)

A2-T2-1-16: CAGTTTGAGCTCGATGCCCATCCCGGGTTCGGGCTCCGCAGCGAG------GGTTTACCTGTGTAACAGACCGCTGTGGC (-6 bp)

A2-T2-1-63: CAGTTTGAGCTCGATGCCCATCCCGGGTTCGGGCTCCGCGGCGAACGTCTGTTACGCAGGTTTACCTGTGTAACAGACCG (+7 bp)

A2-T2-2-05: CAGTTTGAGCTCGATGCCCATCCCGGGTTCGGGCTCCGCGGCGAGGGA--AGGTATACCTGTGTAACAGACCGCTGTGGC (-2 bp)

A2-T2-2-68: CAGTTTGAGCTCGATGCCCATCCCGGGTTCGGGCTCCGCGGCGAGGGTTCGGGCTCCGCGGCGGCGCAGGTATACCTGTG (+17 bp)

A2-T2-3-11: CAGTTTGAGCTCGATGCCCATCCCGGGTTCGGGCTCCGCGGCG------CAGGTTTACCTGTGTAACAGACCGCTGTGGC (-6 bp)

A2-T2-3-10: CAGTTTGAGCTCGATGCCCATCCCGGGTTCGGGCTCCGCGGCG------CAGGTTTACCTGTGTAACAGACCGCTGTGGC (-6 bp)

A2-T2-3-09: CAGTTTGAGCTCGATGCCCATCCCGGGTTCGGGCTCCGCGGCGAGGG------TTTACCTGTGTAACAGACCGCTGTGGC (-6 bp)

A2-T2-3-13: CAGTTTGAGCTCGATGCCCATCCCGGGTTCGGGCTCCGCGGCG-----GCGGGTTTACCTGTGTAACAGACCGCTGTGGC (-5 bp)

A2-T2-3-14: CAGTTTGAGCTCGATGCCCATCCCGGGTTCGGGCTCCGCGGCGAGGG------TTTACCTGTGTAACAGACCGCTGTGGC (-6 bp)

A2-T2-3-60: CAGTTTGAGCTCGATGCCCATCCCGGGTTCGGGCTCCGGGTTTCTACCCTCGGTCTGTTACGCAGGTTTACCTGTGTAACA (+13 bp)

A2-T2-3-01: CAGTTTGAGCTCGATGCCCATC-----------------------CGCAGGTATACCTGTGTAACAGACCGCTGTGGCTCA (-23 bp)

A2-T2-3-08: G------------------------------------------------------TACCTGTGTAACAGACCGCTGTGGCT (-54 bp)

A2-T2-4-17: CAGTTTGAGCTCGATGCCCATCCCGGGTTCGGGCTCCGCGGCGAGGG------TATACCTGTGTAACAGACCGCTGTGGC (-6 bp)

A2-T2-5-23: CAGTTTGAGCTCGATGCCCATCCCGGGTTCGGGCTCCGCGGCGAGGGTATACACAGGTATACCTGTGTAACAGACCGCTGT (+4 bp)

A2-T2-5-22: CAGTTTGAGCTCGATGCCCATCCCGGGTTCGGGCTCCGCGGCG------CAGGTATACCTGTGTAACAGACCGCTGTGGCT (-6 bp)

A2-T2-5-51: CAGTTTGAGCTCGATGCCCATCCCGGGTTCGGGCTCCGCGGCGAGGG------TATACCTGTGTAACAGACCGCTGTGGCT (-6 bp)

A2-T2-5-52: CAGTTTGAGCTCGATGCCCATCCCGGGTTCGGGATGCCCATCC----CGCAGGTTTACCTGTGTAACAGACCGCTGTGGCT (-4 bp)

A2-T2-5-37: CAGTTTGAGCTCGATGCCCATCCCGGGTGCCCATTCGGGCTCCGCGG------------TTTACCTGTGTAACAGACCGCT (-12 bp)

A2-T2-5-20: CAGTTTGAGCTCGATGCCCATCCCGGGTGCCCATTCGGGATGCCCATCC----CGCAGGTTTACCTGTGTAACAGACCGCT (-4 bp)

A2-T2-5-24: CAGTTTGAGCTCGATGCCCATCCCGGGTTCGGGCTCCGCGGCGAGGGCGCAGGTATACCTGTGTAACAGACCGCTGTGGC (-28 bp)

A2-T2-5-36: CAGTTTGAGCTCGATGCCCATCCCGGGTTCGG------------------------------GTAACAGACCGCTGTGGCT (-30 bp)

A2-T2-6-07: CAGTTTGAGCTCGATGCCCATCCCGGGT----------------------------------------------------t

caaattccaccgccatcagaccgagatgatcatcaccaaacagggcaggtaagaatgaaaacttgactgtttcactagttt (-52 bp)

A2-T2-6-08: CAGTTTGAGCTCGATGCCCATCCCGGGTTCGGGCTCCGCGGCG------CAGGTTTACCTGTGTAACAGACCGCTGTGGCT (-6 bp)

A2-T2-6-18: CAGTTTGAGCTCGATGCCCATCCCGGGTTCGGGCTCCGCGGCGAGGG------TATACCTGTGTAACAGACCGCTGTGGCT (-6 bp)

A2-T2-6-62: CAGTTTGAGCTCGATGCCCATCCCGGGTTCGGGCTCCGCGGCG------CAGGTTTACCTGTGTAACAGACCGCTGTGGCT (-6 bp)

A2-T2-6-29: CAGTTTGAGCTCGATGCCCATCCCGGGTTCGGGCTCCGCGGCGAGGG------TTTACCTGTGTAACAGACCGCTGTGGCT (-6 bp)

A2-T2-6-61: CAGTTTGAGCTCGATGCCCATCCCGGGTTCGGGCTCCGCGGCGAGGGA-CAGGTTTACCTGTGTAACAGACCGCTGTGGCT (-1 bp)

A2-T2-6-31: CAGTTTGAGCTCGATGCCCATCCCGGGTTCGGGCTCCGCGGCAAGGGG------ATACCTGTGTAACAGACCGCTGTGGCT (-6 bp)

A2-T2-6-28: CAGTTTGAGCTCGATGCCCATCCCGGGTTCGGGCTCCGCGGCGAG------GGTTTACCTGTGTAACAGACCGCTGTGGCT (-6 bp)

A2-T2-6-29: CAGTTTGAGCTCGATGCCCATCCCGGGTTCGGGCTCCGCGGC--------AGGTTTACCTGTGTAACAGACCGCTGTGGCT (-8 bp)

A2-T2-6-30: CAGTTTGAGCTCGATGCCCATCCCGGGTTCGGGCTCCGCGGCGAGGGCGCGTTTTAGAGCTAGAAAGGTTTACCTGTGTAA (+15 bp)

A2-T2-6-26: CAGTTTGAGCTCGATGCCCATCCCGGGTTCGGGCTCCGC-----------AGGTTTACCTGTGTAACAGACCGCTGTGGCT (-11 bp)

A2-T2-6-30: CAGTTTGAGCTCGATGCCCATCCCGGGTTTA----CCTGTGTAA-----CAGGTTTACCTGTGTAACAGACCGCTGTGGCT (-9 bp)

A2-T2-6-31: CAGTTTGAGCTCGATGCCCATCCCGGG---------------------GCAGGTTTACCTGTGTAACAGACCGCTGTGGCT (-21 bp)

A2-T2-6-32: CAGTTTGAGCTCGATGCCCATCCCGGGTTCGGGCTCCGCGGATTATAAAGTATAGGCTATATTAGGCTATACCTGTGTATACC

gtatacctgtgtaacagaccgctgtggctcaaattccaccgccatcagaccgagatgatcATCACCAAACAGGGCAGGTAA (+31 bp)

**Eomesa2-T2 (7 dpf)**

**Reference:**  CAGTTTGAGCTCGATGCCCATCCCGGGTTCGGGCTCCGCGGCGAGGGCGCAGGTATACCTGTGTAACAGACCGCTGTGGC

A2-T2-1-05: CAGTTTGAGCTCGATGCCCATCCCGGGttcgggctccgcggcgaggg------tttacctgtgtaacagaccgctgtggc (-6 bp)

A2-T2-1-07: CAGTTTGAGCTCGATGCCCATCCCGGGttcgggctccgcggcgtatacctgtggtatacttgtgtaacagaccgctgtggc (+1 bp)

A2-T2-1-04: CAGTTTGAGCTCGATGCCCATCCCGGGttcgggctccgcggcgtatacctgtggtatacttgtgtaacagaccgctgtggc (+1 bp)

A2-T2-1-02: CAGTTTGAGCTCGATGCCCATCCCGGGttcgggctccgcggcgtatacctgtggtatacttgtgtaacagaccgctgtggc (+1 bp)

A2-T2-1-01: CAGTTTGAGCTCGATGCCCATCCCGGGttcgggctccgcggcgtatacctgtggtatacttgtgtaacagaccgctgtggc (+1 bp)

A2-T2-1-08: CAGTTTGAGCTCGATGCCCATCCCGGGttcgggctccgcggcgagg--------------gtgtaacagaccgctgtggc (-14 bp)

A2-T2-1-09: CAGTTTGAGCTCGATGCCCATCCCGGGttcg------------------caggtatacctgtgtaacagaccgctgtggct (-18 bp)

A2-T2-1-06: CAGTTTGAGCTCGATGCCCATCCCGGGtttgggttccgcggcgagg-------tttacctgtgtaacagaccgctgtggc (-7 bp)

A2-T2-3-10: CAGTTTGAGCTCGATGCCCATCCCGGGttcgggctccgcggcgagggc--aggtatacctgtgtaacagaccgctgtggc (-2 bp)

A2-T2-3-08: CAGTTTGAGCTCGATGCCCATCCCGGGttcgggctccgcggcgagggc--aggtatacctgtgtaacagaccgctgtggc (-2 bp)

A2-T2-3-05: CAGTTTGAGCTCGATGCCCATCCCGGGttcgggctccgcggcgagggc--aggtatacctgtgtaacagaccgctgtggc (-2 bp)

A2-T2-3-07: CAGTTTGAGCTCGATGCCCATCCCGGGttcgggctccgcggcggtctgttacgcaggtatacctgtgtaacagaccgctg (+4 bp)

A2-T2-3-06: CAGTTTGAGCTCGATGCCCATCCCGGGttcgggctccgcggc--------aggtatacctgtgtaacagaccgctgtggc (-8 bp)

A2-T2-3-01: CAGTTTGAGCTCGATGCCCATCCCGGGttcgggctccgcggcg------caggtatacctgtgtaacagaccgctgtggc (-6 bp)

A2-T2-3-09: CAGTTTGAGCTCGATGCCCATCCCGGGttcgggctccgcggcgagg-------tttacctgtgtaacagaccgctgtggc (-7 bp)

A2-T2-3-04: CAGTTTGAGCTCGATGCCCATCCCGGGttcgggctccgc-----------aggtttacctgtgtaacagaccgctgtggc (-11 bp)

A2-T2-4-08: CAGTTTGAGCTCGATGCCCATCCCGGGttcgggctccgcggcgagcggcgcaggtttacctgtgtaacagaccgctgtg (+1 bp)

A2-T2-4-03: CAGTTTGAGCTCGATGCCCATCCCGGGttcgggctccgcggcgagcggcgcaggtttacctgtgtaacagaccgctgtg (+1 bp)

A2-T2-4-02: CAGTTTGAGCTCGATGCCCATCCCGGGttcgggctccgcggc---tccgcaggtttacctgtgtaacagaccgctgtggc (-3 bp)

A2-T2-4-06: CAGtttgagctcgatgcccatcccgggccca---tcc----------cgcaggtttacctgtgtaacagaccgctgtggc (-13 bp)

A2-T2-4-05: CAGTTTGAGCTCGATGCCCATCCCGGGttcgggctccgcgggtaggg-----------ctgggtaacagagcgctgtggc (-11 bp)

A2-T2-4-01: CGGCTATCAGTTTGGACAGGGTCCCGGGTGCCTGTACCCGTC--ATTTTAGAGCTAG---------AAATACGATGTA---

---------AGCAGAAACGCAGGTAT ACCTGTGTAACAGACCGCTGTGGCT (-32 bp)

A2-T2-4-04: CAGTTTGAGCTCGATGCCCAT-------------T------------CGCAGGTTTACCTGTGTAACAGACCGCTGTGGCT (-25 bp)

**C T3 site**

**Eomesa1-T3 (24 hpf)**

**Reference**: GAGTTTTAATATCACTGGACTGAACCTGACGGCGCATTATAACGTGTTTGTGGAGGTTGTTCTGGCCGATCCGAACCACT

A1-T3-1-41: GAGTTTTAATATCACTGGACTGAACCTGACGGCGCATTATAACGTG-TTGTGGAGGTTGTTCTGGCCGATCCGAACCACT (-1 bp)

A1-T3-1-42: GAGTTTTAATATCACTGGACTGAACCTGACGGCGCATTATAACG----TGTGGAAGTTGTTCTGGCCGATCCGAACCACT (-4 bp)

A1-T3-1-26: GAGTTTTAATATCACTGGACTGAACCTGACGGCGCATTATAACGTGTGTTGTGGAGGTTGTTCTGGCCGATCCGAACCAC (+1 bp)

A1-T3-2-29: GAGTTTTAATATCACTGGACTGAACCTGACGGCGCATTATAAC--GTTTGTGGAGGTTGTTCTGGCCGATCCGAACCACT (-2 bp)

A1-T3-2-30: GAGTTTTAATATCACTGGACTGAACCTGACGGCGCATTATAACG----TGTGGAGGTTGTTCTGGCCGATCCGAACCACT (-4 bp)

A1-T3-3-53: GAGTTTTAATATCACTGGACTGAACCTGACGGCGCATTATAACG----TGTGGAGGTTGTTCTGGCCGATCCGAACCAC (-4 bp)

A1-T3-3-32: GAGTTTTAATATCACTGGACTGAACCTGACGGCGCATTATAACG----TGTGGAGGTTGTTCTGGCCGATCCGAACCAC (-4 bp)

A1-T3-3-68: GAGTTTTAATATCACTGGACTGAACCTGACGGCGCATTATAACG----TGTGGAGGTTGTTCTGGCCGATCCGAACCAC (-4 bp)

A1-T3-3-52: GAGTTTTAATATCACTGGACTGAACCTGACGGCGCATTATAACG----TGTGGAGGTTGTTCTGGCCGATCCGAACCAC (-4 bp)

A1-T3-4-56: GAGTTTTAATATCACTGGACTGAACCTGACGGCGCATTATAACG----TGTGGAGGTTGTTCTGGCCGATCCGAACCAC (-4 bp)

A1-T3-4-54: GAGTTTTAATATCACTGGACTGAACCTGACGGCGCATTATAACGTGTTTTTTGTATTGTGGAGGTTGTTCTGGCCGATCC (+8 bp)

A1-T3-4-62: GAGTTTTAATATCACTGGACTGAACCTGACGGCGCATTATAACG----TGTGGAGGTTGTTCTGGCCGATCCGAACCAC (-4 bp)

A1-T3-4-34: GAGTTTTAATATCACTGGACTGAACCTGACGGCGCATTATAACGTGTGTTGTGGAGGTTGTTCTGGCCGATCCGAACCA (+1 bp)

A1-T3-4-55: GAGTTTTAATATCACTGGACTGAACCTGACGGCGCATTATAACGTGTGGAGGTTGTGGAGGTTGTTCTGGCCGATCCGA (+5 bp)

A1-T3-4-59: GAGTTTTAATATCACTGGACTGAACCTGACGGCGCATTATAACG----TGTGGAGGTTGTTCTGGCCGATCCGAACCAC (-4 bp)

A1-T3-4-61: GAGTTTTAATATCACTGGACTGAACCTGACGGCGCATTATAAC------GTGGAGGTTGTTCTGGCCGATCCGAACCAC (-6 bp)

A1-T3-5-37: GAGTTTTAATATCACTGGACTGAACCTGACGGCGCATTATAAC--GTTTGTGGAGGTTGTTCTGGCCGATCCGAACCAC (-2 bp)

A1-T3-5-61: GAGTTTTAATATCACTGGACTGAACCTGACGGCGCATTATAACG----TGTGGAGGTTGTTCTGGCCGATCCGAACCAC (-4 bp)

A1-T3-5-38: GAGTTTTAATATCACTGGACTGAACCTGACGGCGCATTATAACG----TGTGGAGGTTGTTCTGGCCGATCCGAACCAC (-4 bp)

A1-T3-5-84: GAGTTTTAATATCACTGGACTGAACCTGACGGCGCATTATAACG----TGTGGAGGTTGTTCTGGCCGATCCGAACCAC (-4 bp)

A1-T3-5-63: GAGTTTTAATATCACTGGACTGAACCTGACGGCGCATTATAACG----TGTGGAGGTTGTTCTGGCCGATCCGAACCAC (-4 bp)

A1-T3-5-40: GAGTTTTAATATCACTGGACTGAACCTGACGGCGCATTATAAC------GTGGAGGTTGTTCTGGCCGATCCGAACCAC (-6 bp)

A1-T3-5-85: GAGTTTTAATATCACTGGACTGAACCTGACGGCGCATTATAAC------GTGGAGGTTGTTCTGGCCGATCCGAACCAC (-6 bp)

A1-T3-5-62: GAGTTTTAATATCACTGGACTGAACCTGACGGCGCATTATAACG----TGTGGAGGTTGCTCTGGCCGATCCGAACCAC (-4 bp)

A1-T3-5-66: GAGTTTTAATATCACTGGACTGAACCTGACGGCGCATTATAACGTGTGGAGGTTGTGGAGGTTGTTCTGGCCGATCCGA (+5 bp)

A1-T3-5-65: GAGTTTTAATATCACTGGACTGAACCTGACGGCGCATTATAACGTGTT--ATAATGTTGTTCTGGCCGATCCGAACCAC (-2 bp)

A1-T3-5-83: GAGTTTTAATATCACTGGACTGAACCTGACGGCGCATTATAACGTGGAGGTGGAGGTTGTGGAGGTTGTGGAGGTTGTT (+18 bp)

A1-T3-5-64: GAGTTTTAATATCACTGGACTGAACCTGACGGCGCATTATAAC------GTGGAGGTTGTTCTGGCCGATCCGAACCAC (-6 bp)

A1-T3-5-60: GAGTTTTAATATCACTGGACTGAACCTGACGGCGCATTATAACGTGTATATAGCAGAATAACACGTCCACCCCCGCGT

GGAGGTTGTGGAGGTTGTTCTGGCCGATCCGAACCACTGGAggtttcagggcggaaaatgggtcacctgtgggaaag (+36 bp)

A1-T3-6-59: GAGTTTTAATATCACTGGACTGAACCTGACGGCGCATTATAACG----TGTGGAGGTTGTTCTGGCCGATCCGAACCAC (-4 bp)

A1-T3-6-43: GAGTTTTAATATCACTGGACTGAACCTGACGGCGCATTATAACGTGT---------TTGTTCTGGCCGATCCGAACCAC (-9 bp)

**Eomesa1-T3 (7 dpf)**

**Reference:**  GAGTTTTAATATCACTGGACTGAACCTGACGGCGCATTATAACGTGTTTGTGGAGGTTGTTCTGGCCGATCCGAACCACT

A1-T3-5-14:  GAGTTTTAATATCACTGGACTGAACCTGACGGCGCATTATAACGTG-TTGTGGAGGTTGTTCTGGCCGATCCGAACCACT (-1 bp)

A1-T3-5-13: GAGTTTTAATATCACTGGACTGAACCTGACGGCGCATTATAACGTG-TTGTGGAGGTTGTTCTGGCCGATCCGAACCACT (-1 bp)

A1-T3-5-09: GAGTTTTAATATCACTGGACTGAACCTGACGACGCATTATAACGTG-TTGTGGAGGTTGTTCTGGCCGATCCGAACCACT (-1 bp)

A1-T3-5-17: GAGTTTTAATATCACTGGACTGAACCTGACGGCGCATTATAACG----TGTGGAGGTTGTTCTGGCCGATCCGAACCACT (-4 bp)

A1-T3-5-07: GAGTTTTAATATCACTGGACTGAACCTGACGGCGCATTATAACG----TGTGGAGGTTGTTCTGGCCGATCCGAACCACT (-4 bp)

A1-T3-5-02: GAGTTTTAATATCACTGGACTGAACCTGACGGCGCATTATAACG----TGTGGAGGTTGTTCTGGCCGATCCGAACCACT (-4 bp)

A1-T3-5-18: GAGTTTTAATATCACTGGACTGAACCTGACAGCGCATTATAACG----TGTGGAGGTTGTTCTGGCCGATCCGAACCACT (-4 bp)

A1-T3-5-11: GAGTTTTAATATCACTGGACTGAACCTGACGGCGCATTATAACG----TGTGGAGGTTGTTCTGGCCGATCCGAACCACT (-4 bp)

A1-T3-5-01: GAGTTTTAATATCACTGGACTGAACCTGACGGCGCATTATAACGTGCGCCGTCAGGTTGTTCTGTTTGTGGAGGTTGTTC (+18 bp)

A1-T3-5-10: GAGTTTTAATATCACTGGACTGAACCTGACGGCGCA-----------TTGTGGAGGTTGTTCTGGCCGATCCGAACCACT (-11 bp)

A1-T3-5-05: GAGTTTTAATATCACTGGACTGAACCTGACGGCGCATTATAACGTGCGCCGTCAGGTTGTTCTGTTTGTGGAGGTTGTTC (+18 bp)

A1-T3-6-17: GAGTTTTAATATCACTGGACTGAACCTGACGGCGCATTATAACG----TGTGGAGGTTGTTCTGGCCGATCCGAACCACT (-4 bp)

A1-T3-6-10: GAGTTTTAATATCACTGGACTGAACCTGACGGCGCATTATAACG----TGTGGAGGTTGTTCTGGCCGATCCGAACCACT (-4 bp)

A1-T3-6-18: GAGTTTTAATATCACTGGACTGA acctgacggcgcattataacgtgcgaggttgtggaggttgttctggccgatccgaa (+4 bp)

A1-T3-6-13: GAGTTTTAATATCACTGGACTGAacctgacggcgcattataacg----tgtggaggttgttctggccgatccgaaccact (-4 bp)

A1-T3-6-11: GAGTTTTAATATCACTGGACTGAacctgacggcgcattataacg----tgtggaggttgttctggccgatccgaaccact (-4 bp)

A1-T3-6-05: GAGTTTTAATATCACTGGACTGAacctgacggcgcattataacg-----gtggaggttgttctggccgatccgaaccact (-5 bp)

A1-T3-6-15: GAGTTTTAATATCACTGGACTGAacctgacggcgcattataacggcgcattgtggaggttgttctggccgatccgaacca (+2 bp)

A1-T3-6-08: GAGTTTTAATATCACTGGACTGAacctgacggcgcattataac------gtggaggttgttctggccgatccgaaccact (-6 bp)

A1-T3-6-07: GAGTTTTAATATCACTGGACTGAacctgacggcgcattataac------gtggaggttgttctggccgatccgaaccact (-6 bp)

A1-T3-6-19: GAGTTTTAATATCACTGGACTGAacctgacggcgcattataacgtggaggttgtggaggttgttctggccgatccgaacc (+3 bp)

A1-T3-6-06: GAGTTTTAATATCACTGGACTGAACCTGACGGCGCATTATAACGTTTTACATTATAACGAGGCCTTGTGGAGGTTGTTCTG (+17 bp)

A1-T3-7-13: GAGTTTTAATATCACTGGACTGAACCTGACGGCGCATTATAACG----TGTGGAGGTTGTTCTGGCCGATCCGAACCACT (-4 bp)

A1-T3-7-07: GAGTTTTAATATCACTGGACTGAACCTGACGGCGCATTATAACG----TGTGGAGGTTGTTCTGGCCGATCCGAACCACT (-4 bp)

A1-T3-7-11: GAGTTTTAATATCACTGGACTGAACCTGACGGCGCATTATAACGTGTGGAGGTTGTGGAGGTTGTTCTGGCCGATCCGAA (-5 bp)

**Eomesa2-T3 (24 hpf)**

**Reference:** GAGTTTTAATATCACCGGACTGAACCTGACGGCGCATTATAACGTGTTTGTGGAGGTTGTTCTGGCCGATCCTAACCACT

A2-T3-1-26: GAGTTTTAATATCACCGGACTGAACCTGACGGCGCATTATAACG----TGTGGAGGTTGTTCTGGCCGATCC TAACCAC (-4 bp)

A2-T3-1-95: GAGTTTTAATATCACCGGACTGAACCTGACGGCGCATTATAACG----TGTGGAGGTTGTTCTGGCCGATCCTAACCAC (-4 bp)

A2-T3-1-36: GAGTTTTAATATCACCGGACTGAACCTGACGGCGCATTATAACG----TGTGGAGGTTGTTCCGGCCGATCCTAACCAC (-4 bp)

A2-T3-1-33: GAGTTTTAATATCACCGGACTGAACCTGACGGCGCATTATAACG----TGTGGAGGTTGTTCTGGCCGATCCTAACCAC (-4 bp)

A2-T3-1-25: GAGTTTTAATATCACCGGACTGAACCTGACGGCGCATTA--------TTGTGGAGGTTGTTCTGGCCGATCCTAACCAC (-8 bp)

A2-T3-1-01: GAGTTTTAATATCACCGGACTGAACCTGACGGCGCATTATAAC------GTGGAGGTTGTTCTGGCCGATCCTAACCAC (-6 bp)

A2-T3-2-53: GAGTTTTAATATCACCGGACTGAACCTGACGGCGCATTATAACG----TGTGGAGGTTGTTCTGGCCGATCCTAACCAC (-4 bp)

A2-T3-4-51: GAGTTTTAATATCACCGGACTGAACCTGACGGCGCATTATAAC------GTGGAGGTTGTTCTGGCCGATCCTAACCAC (-6 bp)

A2-T3-4-46: GAGTTTTAATATCACCGGACTGAACCTGACGGCGCATTATAACGTGGAGGTTGTGGAGGTTGTTCTGGCCGATCCTAAC (+3 bp)

A2-T3-4-06: GAGTTTTAATATCACCGGACTGAACCTGACGGCGCATTATAACGGCGCATTATAATTGTGGAGGTTGTTCTGGCCGATC (+8 bp)

A2-T3-4-07: GAGTTTTAATATCACCGGACTGAACCTGACGGCGCATTATAACGTGTTTTGTTATATATATTTGTGGAGGTTGCTCTGGC (+14 bp)

A2-T3-4-49: GAGTTTTAATATCACCGGACTGAACCTGACGGCGCATTATAACGTGTAACATCATATTTTAATTATAACGTGTAACATCA

TATTTTTGTGGAGGTTGTTCTGGCCGATCCTAACCACTGGAGATTTCAGGGCGGAAAATGGGTCACCTGCGGGAAAGCG (+38 bp)

A2-T3-4-47: GAGTTTTAATATCACCGGACTGAACCTGACGGCGCATTATAACGTGTAACATCATATTTTAATTATAACGTGTAACATCA

TATTTTTGTGGAGGTTGTTCTGGCCGATCCTAACCACTGGAGATTTCAGGGCGGAAAATGGGTCACCTGCGGGAAAGCG (+38 bp)

A2-T3-4-48: GAGTTTTAATATCACCGGACTGAACCTGACGGCGCATTATAACG------------------------ATCCTAACCACT (-24 bp)

A2-T3-4-50: GAGTTTTAATATCACCGGACTGAACCTGACGGCGCATTATAACGTGTAACATCATATTTTAATTATAACGTGTAACATCA

TATTTTTGTGGAGGTTGTTCTGGCCGATCCTAACCACTGGAGATTTCAGGGCGGAAAATGGGTCACCTGCGGGAAAGCG (+38 bp)

A2-T3-5-12: GAGTTTTAATATCACCGGACTGAACCTGACGGCGCATTATAACGTTATAGTTTGTGGAGGTTGTTCTGGCCGATCCTAAC (+4 bp)

A2-T3-5-42: GAGTTTTAATATCACCGGACTGAACCTGACGGCGCATTATAACGTG-TTCTGGAGGTTGTTCTGGCCGATCCTAACCACT (-1 bp)

A2-T3-5-55: GAGTTTTAATATCACCGGACTGAACCTGACGGCGCATTATAACG---TTGTGGAGGTTGTTCTGGCCGATCCTAACCACT (-3 bp)

A2-T3-5-41: GAGTTTTAATATCACCGGACTGAACCTGACGGCGCATTATAACG----TGTGGAGGTTGTTCTGGCCGATCCTAACCACT (-4 bp)

A2-T3-5-44: GAGTTTTAATATCACCGGACTGAACCTGACGGCGCATTATGACGTG-TTGTGGAGGTTGTTCTGGCCGATCCTAACCACT (-1 bp)

A2-T3-5-11: GAGTTTTAATATCACCGGACTGAACCTGACGGCGCATTATAACG----TGTGGAGGTTGTTCTGGCCGATCCTAACCACT (-4 bp)

A2-T3-5-08: GAGTTTTAATATCACCGGACTGAACCTGACGGCGCATTATAACG----TGTGGAGGTTGTTCTGGCCGATCCTAACCACT (-4 bp)

A2-T3-5-53: GAGTTTTAATATCACCGGACTGAACCTGACGGCGCATTATAACG----TGTGGAGGTTGTTCTGGCCGATCCTAACCACT (-4 bp)

A2-T3-5-09: GAGTTTTAATATCACCGGACTAAACCTGACGGCGCATTATAACGTGGAGGTTGTGGAGGTTGTTCTGGCCGATCCTAACC (+3 bp)

A2-T3-5-54: GAGTTTTAATATCACCGGACTGAACCTGACGGCGCATTATAACG----TGTGGAGGTTGTTCTGGCCGATCCTAACCACT (-4 bp)

A2-T3-5-56: GAGTTTTAATATCACCGGACTGAACCTGACGGCGCATTATAAC------GTGGAGGTTGTTCTGGCCGATCCTAACCACT (-6 bp)

A2-T3-5-10: GAGTTTTAATATCACCGGACTGAACCTGACGGCGCATTATAAC------GTGGAGGTTGTTCTGGCCGATCCTAACCCCT (-6 bp)

A2-T3-5-52: GAGTTTTAATATCACCGGACCGAACCTGACGGCGCATTATAACGTGTTCTGTTAACGTGTTCATTGTGGAGGTTGTTCTG (+16 bp)

A2-T3-5-40: GAGTTTTAACATCACCGGACTGAACCTGACGGCGCATTATAAC------GTGGAGGTTGTTCTGGCCGATCCTAACCACT (-6 bp)

A2-T3-6-75: GAGTTTTAATATCACCGGACTGAACCTGACGGCGCATTATAACGTG-TTGTGGAGGTTGTTCTGGCCGATCCTAACCACT (-1 bp)

A2-T3-6-14: GAGTTTTAATATCACCGGACTGAACCTGACGGCGCATTATAACGTG-TTGTGGAGGTTGTTCTGGCCGATCCTAACCACT (-1 bp)

A2-T3-6-60: GAGTTTTAATATCACCGGACTGAATCTGACGGCGCATTATAACGTG-TTGTGGAGGTTGTTCTGGCCGATCCTAACCACT (-1 bp)

A2-T3-6-73: GAGTTTTAATATCACCGGACTGAACCTGACGGCGCATTATAACG----TGTGGAGGTTGTTCTGGCCGATCCTAACCACT (-4 bp)

A2-T3-6-64: GAGTTTTAATATCACCGGACTGAACCTGACGGCGCATTATAACGTGT------AGGTTGTTCTGGCCGATCCTAACCACT (-6 bp)

A2-T3-6-15: GAGTTTTAATATCACCGGACTGAACCTGACGGCGCATTATAAC------GTGGAGGTTGTTCTGGCCGATCCTAACCACT (-6 bp)

A2-T3-6-58: GAGTTTTAATATCACCGGACTGAACCTGACGGCGCATTATAACGTG--TATGGAGGTTGTTCTGGCCGATCCTAACCACT (-2 bp)

A2-T3-6-63: GAGTTTTAATATCACCGGACTGAACCTGACGGCGCATTATAAC--------GGAGGTTGTTCTGGCCGATCCTAACCACT (-8 bp)

A2-T3-6-10: GAGTCTTAATATCACCGGACTGAACCTGACGGCGCATTGCTATTTCTAGCTCTAAAAGGCGCATTATAACGTGTTTGGTTT

TAGAGCTAGAAATAGCAAGTTAAAATAAGGCTAGTCCGTTATCAATTGTGGAGGTTGTTCTGGCCGATCCTAACCACTGG (+79 bp)

**Eomesa2-T3 (7 dpf)**

**Reference:** GAGTTTTAATATCACCGGACTGAACCTGACGGCGCATTATAACGTGTTTGTGGAGGTTGTTCTGGCCGATCCTAACCACT

A2-T3-5-08: GAGTTTTAATATCACCGGACTGAACCTGACGGCGCATTATAACGTGTGGAGGTTGTGGAGGTTGTTCTGGCCGATCCTAA (+5 bp)

A2-T3-5-12: GAGTTTTAATATCACCGGACTGAACCTGACGGCGCATTATAACG----TGTGGAGGTTGTTCTGGCCGATCCTAACCACTG (-4 bp)

A2-T3-5-07: GAGTTTTAATATCACCGGACTGAACCTGACGGCGCATTATAACG----TGTGGAGGTTGTTCTGGCCGATCCTAACCACTG (-4 bp)

A2-T3-5-09: GAGTTTTAATATCACCGGACTGAACCTGACGGCGCATTATAACG----TGTGGAGGTTGTTCTGGCCGATCCTAACCACTG (-4 bp)

A2-T3-5-11: GAGTTTTAATATCACCGGACTGAACCTGACGGCGCATTATAACG----TGTGGAGGTTGTTCTGGCCGATCCTAACCACTG (-4 bp)

A2-T3-5-01: GAGTTTTAATATCACCGGACTGAACCTGACGGCGCATTATAACGGCGCATTGTGGAGGTTGTTCTGGCCGATCCTAACCAC (+2 bp)

A2-T3-5-06: GAGTTTTAATATCACCGGACTGAACCTGACGGCGCATTATAACGCATTATAACGTATCGCATTATAACGTATCCTTGTGGAG (+27 bp)

A2-T3-6-10: GAGTTTTAATATCACCGGACTGAACCTGACGGCGCATTATAACGTG-TTGTGGAGGTTGTTCTGGCCGATCCTAACCACTG (-1 bp)

A2-T3-6-06: GAGTTTTAATATCACCGGACTGAACCTGACGGCGCATTATAACG----TGTGGAGGTTGTTCTGGCCGATCCTAACCACTG (-4 bp)

A2-T3-6-12: GAGTTTTAATATCACCGGACTGAACCTGACGGCGCATTATAACG----TGTGGAGGTTGTTCTGGCCGATCCTAACCACTG (-4 bp)

A2-T3-6-08: GAGTTTTAATATCACCGGACTGAACCTGACGGCGCATTATAACGTGGAGGTTGTGGAGGTTGTTCTGGCCGATCCTAACCA (+3 bp)

A2-T3-6-04: GAGTTTTAATATCACCGGACTGAACCTGACGGCGCATTATAACGTGGAGGTTGTGGAGGTTGTTCTGGCCGATCCTAACCA (+3 bp)

A2-T3-7-01: GAGTTTTAATATCACCGGACTGAACCTGACGGCGCATTATAACG----TGTGGAGGTTGTTCTGGCCGATCCTAACCACTG (-4 bp)

A2-T3-7-03: GAGTTTTAATATCACCGGACTGAACCTGACGGCGCATTATAAC------GTGGAGGTTGTTCTGGCCGATCCTAACCACTG (-6 bp)

A2-T3-7-09: GAGTTTTAATATCACCGGACTGAACCTG-------------------TTGTGGAGGTTGTTCTGGCCGATCCTAACCACTGC (-19 bp)

**D T4 site**

**Eomesa1-T4 (24 hpf)**

**Reference:** gGGTCCAGGTATTCGTCGTCTCTGCATTACGGCTCGGTTCTTCCGCCCGCCGGGTTCTCCTCCGCCGTGTGCGCCGGTCGCA

A1-T4-3-91: gGGTCCAGGTATTCGTCGTCTCtgcattacggctcggttcttccgccgagctccgacgtgttctcgccgggttctcctccgc (+17 bp)

A1-T4-4-70: gGGTCCAGGTATTCGTCGTCTCtgcattacggctcggtt-----------------ctcctccgccgtgtgcgccggtcgcag (-17 bp)

A1-T4-5-73: gGGTCCAGGTATTCGTCGTCTCtgcattacggctcggttcttccgcc----gagttctcctccgccgtgtgcgccggtcgca (-4 bp)

A1-T4-5-83: gGGTCCAGGTATTCGTCGTCTCtgcattacggctcggttcttccgcgctgtatgcgcgccgggttctcctccgccgtgtgcg (+9 bp)

A1-T4-5-82: gGGTCCAGGTATTCGTCGTCTCtgcattacggctcggtt-----------------ctcctccgccgtgtgcgccggtcgcag (-17 bp)

A1-T4-6-50: gGGtccaggtattcgtcgtctctgcattacggctcggttcttccggcgggcgccgggttctcctccgccgtgtgcgccggt (+3 bp)

**Eomesa1-T4 (7 dpf)**

**Reference:** GGGTCCAGGTATTCGTCGTCTCTGCATTACGGCTCGGTTCTTCCGCCCGCCGGGTTCTCCTCCGCCGTGTGCGCCGGTCG

A1-T4-4-01: GGGTCCAGGTATTCGTCGTCTCtgcattacggctcggttcttccg-------ggttctcctccgccgtgtgcgccggtcg (-7 bp)

A1-T4-4-06: GGGTCCAGGTATTCGTCGTCTCtgcattacggctcggttctt-------ccgggttctcctccgccgtgtgcgccgggcg (-7 bp)

A1-T4-9-15: GGGTCCAGGTATTCGTCGTCTCtgcattacggctcggttcttccgc----cgggttctcctccgccgtgtgcgccggtcg (-4 bp)

A1-T4-9-11: GGGTCCAGGTATTCGTCGTCTCtgcattacggctcggttcttccgccag----gttctcctccgccgtgtgcgccggtcg (-4 bp)

**Eomesa2-T4 (24 hpf)**

**Reference:** GGGTCCAGGTATTCGTCGTCTCTGCATTACGGCTCGGTTCTTCCGCCCGCGGGGTTCTCCTCCGCCGTGTGCGCCAGTCGCA

A2-T4-2-12: gggtccaggtattcgtcgtctctgcattacggctcggttcttccgc----ggggttctCCTCCGCCGTGTGCGCCAGTCGCA (-4 bp)

A2-T4-2-74: gggtccaggtattcgtcgtctctgcgttacggctcggttcttccg------gggttctCCTCCGCCGTGTGCGCCAGTCGCA (-6 bp)

A2-T4-3-11: gggtccaggtattcgtcgtctctgcattacggctcggttcttccgc----ggggttctCCTCCGCCGTGTGCGCCAGTCGCA (-4 bp)

A2-T4-4-16: gggtccaggtattggttgtgtatgcagtatggctcggttcttccgc----ggggttctCCTCCGCCGTGTGCGCCAGTCGCA (-4 bp)

A2-T4-4-22: gggtccaggtattcgtcgtctctgcattacggctcggttcttccg-------------------ccgtgtgcGCCAGTCGCAG (-19 bp)

A2-T4-5-37: gggtccaggtattcgtcgtctctgcattacggctcggttcttccgc----ggggttctCCTCCGCCGTGTGCGCCAGTCGCA (-4 bp)

A2-T4-5-89: gggtccaggtattcgtcgtctctgcattacggctcggttcttccg------gggttctCCTCCGCCGTGTGCGCCAGTCGCA (-6 bp)

A2-T4-5-36: GGGTCCAGGTATTCGTCGTCTCTGCATTACGGCTCGGTTCTTCGGTTGGGTCGGTGCCACTTTTTCAAGTTGATAACGGACT

AGCCTTATTTTAACTTGCTATTTCTAGCTCTAAAACGCGGGCGGAAGA-----GCGGGGTTCTCCTCCGCCGTGTGCGCCA (+82 bp)

A2-T4-6-87: GGGTCCAGGTATTCGTCGTCTCTGCATTacggctcggttcttccgcg----gggttctcctccgccgtgtgcgccagtcgca (-4 bp)

**Eomesa2-T4 (7 dpf)**

**Reference:** GGGTCCAGGTATTCGTCGTCTCTGCATTACGGCTCGGTTCTTCCGCCCGCGGGGTTCTCCTCCGCCGTGTGCGCCAGTCG

A2-T4-9-14: GGGTCCAggtattcgtcgtctctgcattacggctcggttcttccgc----ggggttctCCTCCGCCGTGTGCGCCAGTCG (-4 bp)

A2-T4-9-03: GGGTCCAggtattcgtcgtctctgcattacggctcggttcttccgc----ggggttctCCTCCGCCGTGTGCGCCAGTCG (-4 bp)

A2-T4-9-04: GGGTCCAggtattcgtcgtctctgcattacggctcggttcttccgc----ggggttctCCTCCGCCGTGTGCGCCAGTCG (-4 bp)

A2-T4-9-11: GGGTCCAggtattcgtcgtctctgcattacggctcggttcttccgC----ggggttctCCTCCGCCGTGTGCGCCAGTCG (-4 bp)

A2-T4-9-02: GGGTCCAggtattcgtcgtctctgcattacggctcggttcttccgc----ggggttctCCTCCGCCGTGTGCGCCAGTCG (-4 bp)

A2-T4-9-15: GGGTCCAggtattcgtcgtctctgcattacggctcggttcttccgc----ggggttctCCTCCGCCGTGTGCGCCAGTCG (-4 bp)

A2-T4-9-10: GGGTCCAggtattcgtcgtctctgcattacggctcggttcttccgc----ggggttctCCTCCGCCGTGTGCGCCAGTCG (-4 bp)

A2-T4-9-01: GGGTCCAggtattcgtcgtctctgcattacggctcggttcttccgc----ggggttctCCTCCGCCGTGTGCGCCAGTCG (-4 bp)

A2-T4-9-13: GGGTCCAggtattcgtcgtctctgcattacggctcggttcttccgc----ggggttctCCTCCGCCGTGTGCGCCAGTCG (-4 bp)

**S3 Fig. The sequences of the target region of *eomesa* in the common carp larvae at 24 hpf and 7 dpf.**

Notes: The letters highlighted in blue indicate the target region and the letters highlighted in red indicate the PAM region. The letters highlighted in yellow indicate the inserted nucleotides and the dash lines indicate the deleted nucleotides in gene editing fish compared to wild type fish.
