# Supplementary material for: Disruption of T-box transcription factor eomesa results in abnormal development of median fins in Oujiang color common carp Cyprinus carpio
Source: PLoS One. 2023 Mar 2;18(3):e0281297. doi: 10.1371/journal.pone.0281297 (PMC9980737; doi:10.1371/journal.pone.0281297)
Supplement: S3 Table — (DOCX) [file pone.0281297.s006.docx]

**Table S3. The statistics of the knockout efficiency on 24 hpf embryos with three sampling replicates**

|  | Times of Injection | T1 | | | T2 | | | T3 | | | T4 | | |
| --- | --- | --- | --- | --- | --- | --- | --- | --- | --- | --- | --- | --- | --- |
| *eomesa1* | 1 | 6.7% | 42.9% | 40.0% | 26.7% | 13.3% | 46.7% | 13.3% | 14.3% | 30.8% | 0.0% | 0.0% | 6.7% |
|  | 2 | 66.7% | 86.7% | 0.0% | 46.7% | 14.3% | 61.5% | 50.0% | 92.9% | 15.4% | 8.3% | 20.0% | 10.0% |
| *eomesa2* | 1 | 60.0% | 73.3% | 13.3% | 26.7% | 13.3% | 64.3% | 40.0% | 6.7% | 0.0% | 0.0% | 15.4% | 6.7% |
|  | 2 | 40.0% | 73.3% | 6.7% | 6.7% | 46.7% | 93.3% | 53.3% | 86.7% | 62.5% | 20.0% | 20.0% | 10.0% |
