# Supplementary material for: Disruption of T-box transcription factor eomesa results in abnormal development of median fins in Oujiang color common carp Cyprinus carpio
Source: PLoS One. 2023 Mar 2;18(3):e0281297. doi: 10.1371/journal.pone.0281297 (PMC9980737; doi:10.1371/journal.pone.0281297)
Supplement: S5 Table — (DOCX) [file pone.0281297.s008.docx]

**Table S5. The statistics of the mutation types on 24 hpf embryos**

| **Gene** | **T1** | | | | **T2** | | | | **T3** | | | | **T4** | | | |
| --- | --- | --- | --- | --- | --- | --- | --- | --- | --- | --- | --- | --- | --- | --- | --- | --- |
|  | Mutation  number | In-frame  indels | Frame-shift  indels | Mutation  type number | Mutation  number | In-frame  indels | Frame-shift  indels | Mutation  type number | Mutation  number | In-frame  indels | Frame-shift  indels | Mutation  type number | Mutation  number | In-frame  indels | Frame-shift  indels | Mutation  type number |
| *eomesa1* | 35 | 8 | 27 | 20 | 30 | 12 | 18 | 18 | 31 | 7 | 24 | 10 | 6 | 1 | 5 | 4 |
| *eomesa2* | 38 | 15 | 23 | 21 | 37 | 21 | 16 | 19 | 38 | 11 | 27 | 14 | 9 | 3 | 6 | 5 |
