# Supplementary material for: Disruption of T-box transcription factor eomesa results in abnormal development of median fins in Oujiang color common carp Cyprinus carpio
Source: PLoS One. 2023 Mar 2;18(3):e0281297. doi: 10.1371/journal.pone.0281297 (PMC9980737; doi:10.1371/journal.pone.0281297)
Supplement: S6 Table — (DOCX) [file pone.0281297.s009.docx]

**Table S6. The individual knockout efficiency on 7 dpf larvae with three random individuals**

|  | T1 | | | T2 | | | T3 | | | T4 | | |
| --- | --- | --- | --- | --- | --- | --- | --- | --- | --- | --- | --- | --- |
| *eomesa1* | 70.0% | 100.0% | 80.0% | 80.0% | 60.0% | 90.0% | 100.0% | 100.0% | 55.6% | 20.0% | 0.0% | 20.0% |
| *eomesa2* | 80.0% | 60.0% | 90.0% | 90.0% | 80.0% | 62.5% | 80.0% | 40.0% | 30.0% | 30.0% | 0.0% | 63.6% |
