# Supplementary material for: Disruption of T-box transcription factor eomesa results in abnormal development of median fins in Oujiang color common carp Cyprinus carpio
Source: PLoS One. 2023 Mar 2;18(3):e0281297. doi: 10.1371/journal.pone.0281297 (PMC9980737; doi:10.1371/journal.pone.0281297)
Supplement: S7 Table — (DOCX) [file pone.0281297.s010.docx]

**Table S7. The statistics of the mutation types on 7 dpf larvae**

| **Gene** | **T1** | | | | **T2** | | | | **T3** | | | | **T4** | | | |
| --- | --- | --- | --- | --- | --- | --- | --- | --- | --- | --- | --- | --- | --- | --- | --- | --- |
|  | Mutation  number | In-frame  indels | Frame-shift  indels | Mutation  type number | Mutation  number | In-frame  indels | Frame-shift  indels | Mutation  type number | Mutation  number | In-frame  indels | Frame-shift  indels | Mutation  type number | Mutation  number | In-frame  indels | Frame-shift  indels | Mutation  type number |
| *eomesa1* | 27 | 9 | 18 | 17 | 22 | 9 | 13 | 16 | 25 | 5 | 20 | 11 | 4 | 0 | 4 | 2 |
| *eomesa2* | 25 | 4 | 21 | 13 | 23 | 4 | 19 | 13 | 15 | 4 | 11 | 8 | 9 | 0 | 9 | 1 |
