# Supplementary material for: Disruption of T-box transcription factor eomesa results in abnormal development of median fins in Oujiang color common carp Cyprinus carpio
Source: PLoS One. 2023 Mar 2;18(3):e0281297. doi: 10.1371/journal.pone.0281297 (PMC9980737; doi:10.1371/journal.pone.0281297)

Examination of potential off-target on *eomesb* gene

**Table S9. The primers used for amplifying off-target regions**

| **Gene name** | **Off-target region** | **Forward Primer** | **Reverse Primer** | **Size (bp)** |
| --- | --- | --- | --- | --- |
| *eomesb1* | CACGCATTATAATGTGTTTG | GCTGCTAAACCTGAAATAGTGCC | TTCAGTCCTCCGAACCTTGC | 330 |
| *eomesb2* | CACTCATTATAATGTGTTTG | TTCAGTCCTCCGAACCTTGC | CCTGTGTTCGGGGATTCGG | 264 |

**Table S10. The results of Sanger sequencing chromatographs for 10 larvae at 7 dpf**

| **Larvae ID** | **Chaos position at chromatograph** | |
| --- | --- | --- |
|  | *eomesb1* | *eomesb2* |
| 01 | 90 bp before target site | No chaos |
| 02 | No chaos | 160 bp after target site |
| 03 | 90 bp before target site | No chaos |
| 04 | No chaos | 160 bp after target site |
| 05 | No chaos | No chaos |
| 06 | No chaos | 160 bp after target site |
| 07 | No chaos | No chaos |
| 08 | 90 bp before target site | No chaos |
| 09 | No chaos | No chaos |
| 10 | 90 bp before target site | No chaos |

**Larvae 01**

**PCR sequencing result**


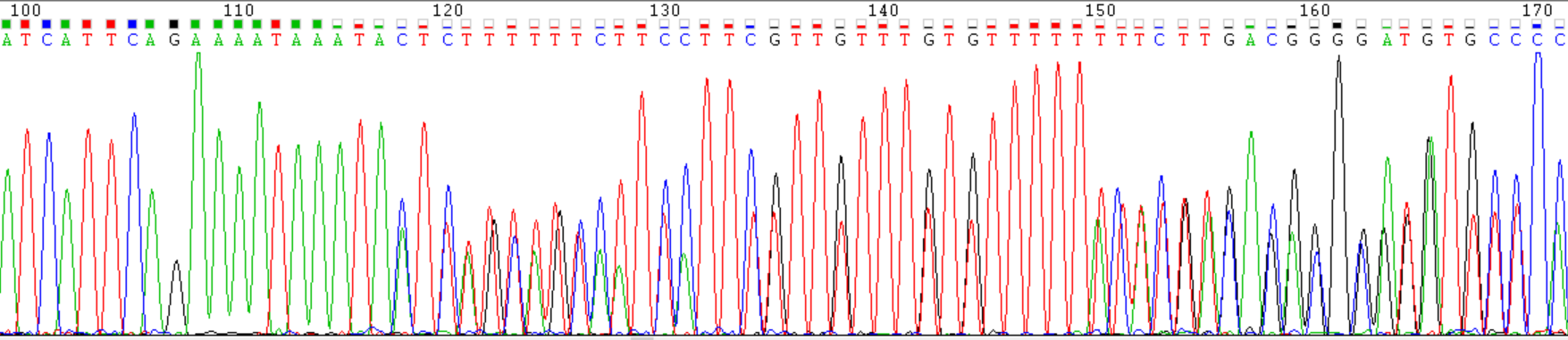

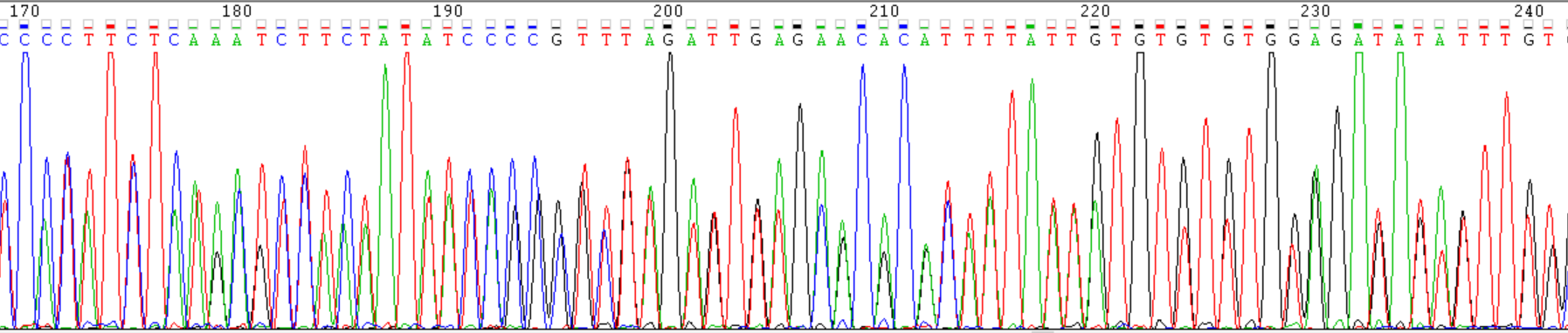


***Eomesb1***

***Eomesb2***


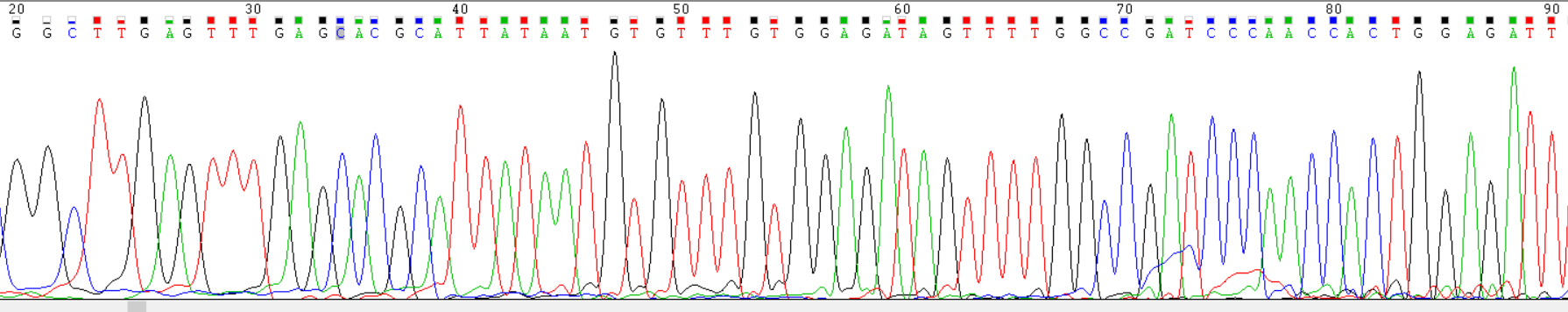

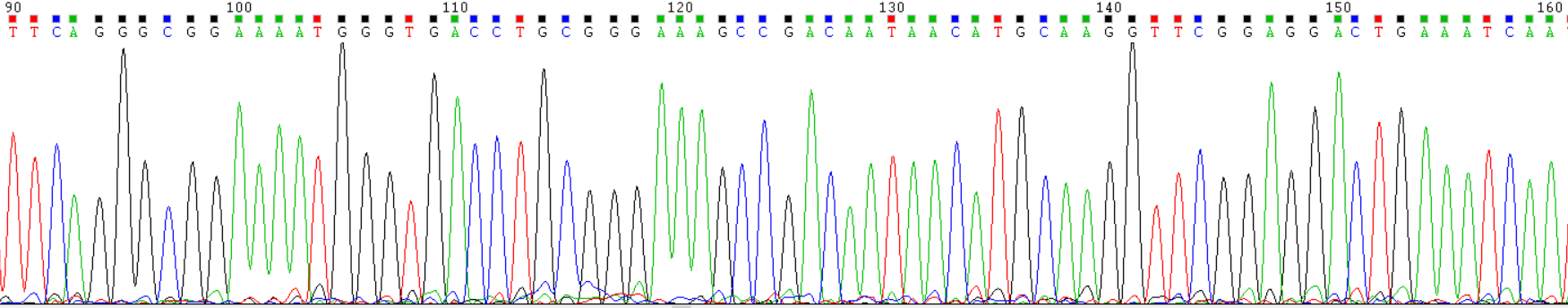

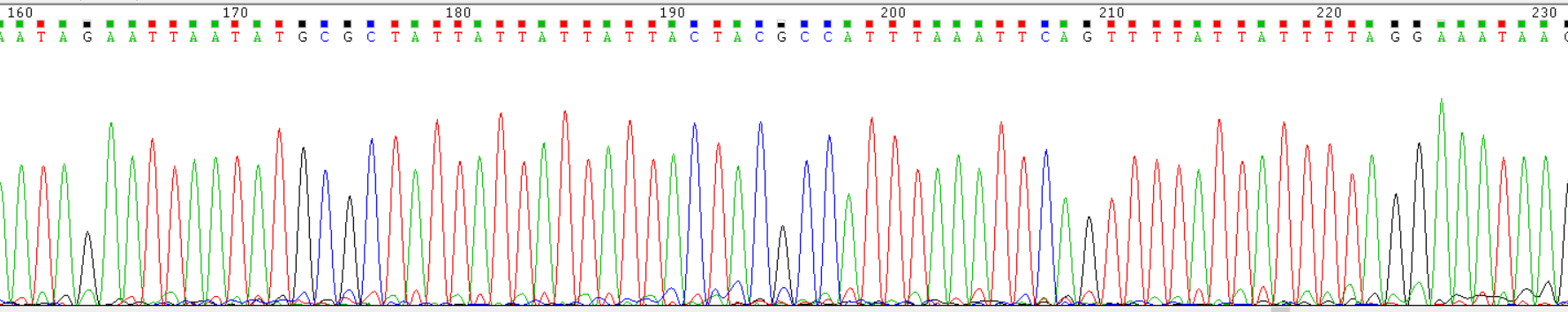


**Larvae 01**

***eomesb1***

**TA clone sequencing result:** **Ten clones with indels among the ten examined clones**

**Reference:** AAAATAAATAATAATTTGCATTCATTCTTTGTTTTTGTTTTTTACTTGACAGGCGGATGTTCCCATT

TCTAAGCTTCAATATTACCGGCTTGAGTTTGAGCACGCATTATAATGTGTTTGTGGAGATAGTTTTGG

B1-1-03: AAAATAAATACTCATTTGCATTCATTCTTTGTTTTTGTTTTTTTACTTGACAGGCGGATGTTCCCATT

TCTAAGCTTCAATATTACCGGCTTGAGTTTGAGCACGCATTATAATGTGTTTGTGGAGATAGTTTTGG (+1 bp)

B1-1-04: AAAATAAATACTCATTTGCATTCATTCTTTGTTTTTGTTTTTTTACTTGACAGGCGGATGTTCCCATT

TCTAAGCTTCAATATTACCGGCTTGAGTTTGAGCACGCATTATAATGTGTTTGTGGAGATAGTTTTGG (+1 bp)

B1-1-05: AAAATAAATACTCATTTGCATTCATTCTTTGTTTTTGTTTTTTTACTTGACAGGCGGATGTTCCCATT

TCTAAGCTTCAATATTACCGGCTTGAGTTTGAGCACGCATTATAATGTGTTTGTGGAGATAGTTTTGG (+1 bp)

B1-1-06: ATA---AATAATTTGCATTCATTCTTTGTTTTTTGTTTTTTTACTTGACAGGCGGATGTTCCCATTTC

TAAGCTTCAATATTACCGGCTTGAGTTTGAGCACGCATTATAATGTGTTTGTGGAGATAGTTTTGG (-1 bp)

B1-1-07: ATA---AATAATTTGCATTCATTCTTTGTTTTTTGTTTTTTTACTTGACAGGCGGATGTTCCCATTTC

TAAGCTTCAATATTACCGGCTTGAGTTTGAGCACGCATTATAATGTGTTTGTGGAGATAGTTTTGG (-1 bp)

B1-1-08: ATA---AATAATTTGCATTCATTCTTTGTTTTTTGTTTTTTTACTTGACAGGCGGATGTTCCCATTTC

TAAGCTTCAATATTACCGGCTTGAGTTTGAGCACGCATTATAATGTGTTTGTGGAGATAGTTTTGG (-1 bp)

B1-1-09: AAAATAAATACTCATTTGCATTCATTCTTTGTTTTTGTTTTTTTACTTGACAGGCGGATGTTCCCATT

TCTAAGCTTCAATATTACCGGCTTGAGTTTGAGCACGCATTATAATGTGTTTGTGGAGATAGTTTTGG (+1 bp)

B1-1-10: ATA---AATAATTTGCATTCATTCTTTGTTTTTTGTTTTTTTACTTGACAGGCGGATGTTCCCATTTC

TAAGCTTCAATATTACCGGCTTGAGTTTGAGCACGCATTATAATGTGTTTGTGGAGATAGTTTTGG (-1 bp)

B1-1-11: ATA---AATAATTTGCATTCATTCTTTGTTTTTTGTTTTTTTACTTGACAGGCGGATGTTCCCATTTC

TAAGCTTCAATATTACCGGCTTGAGTTTGAGCACGCATTATAATGTGTTTGTGGAGATAGTTTTGG (-1 bp)

B1-1-12: AAAATAAATACTCATTTGCATTCATTCTTTGTTTTTGTTTTTTTACTTGACAGGCGGATGTTCCCATT

TCTAAGCTTCAATATTACCGGCTTGAGTTTGAGCACGCATTATAATGTGTTTGTGGAGATAGTTTTGG (+1 bp)

**Larvae 02**

**PCR sequencing result**


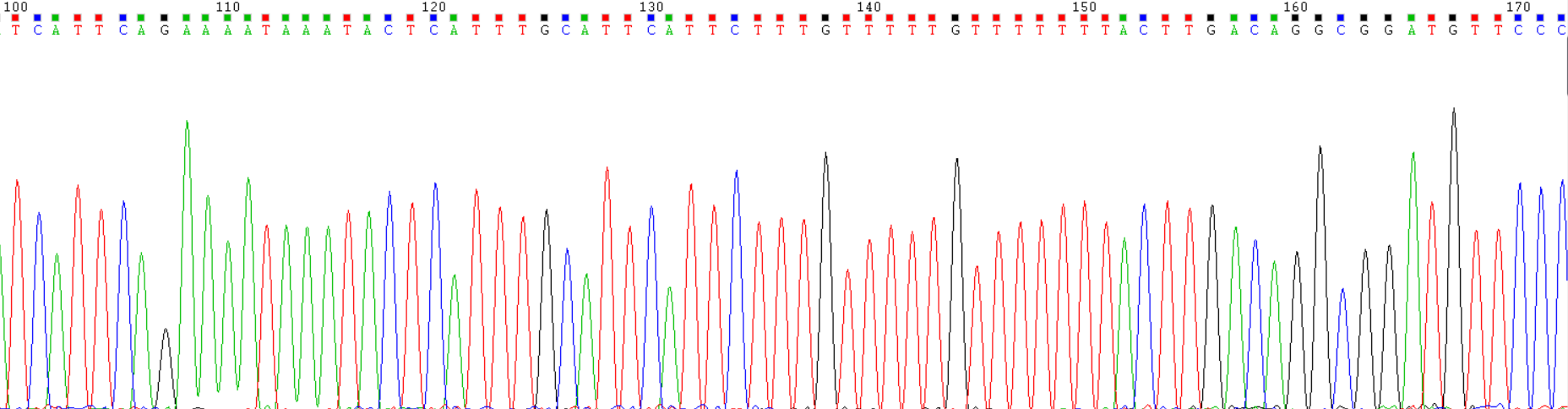

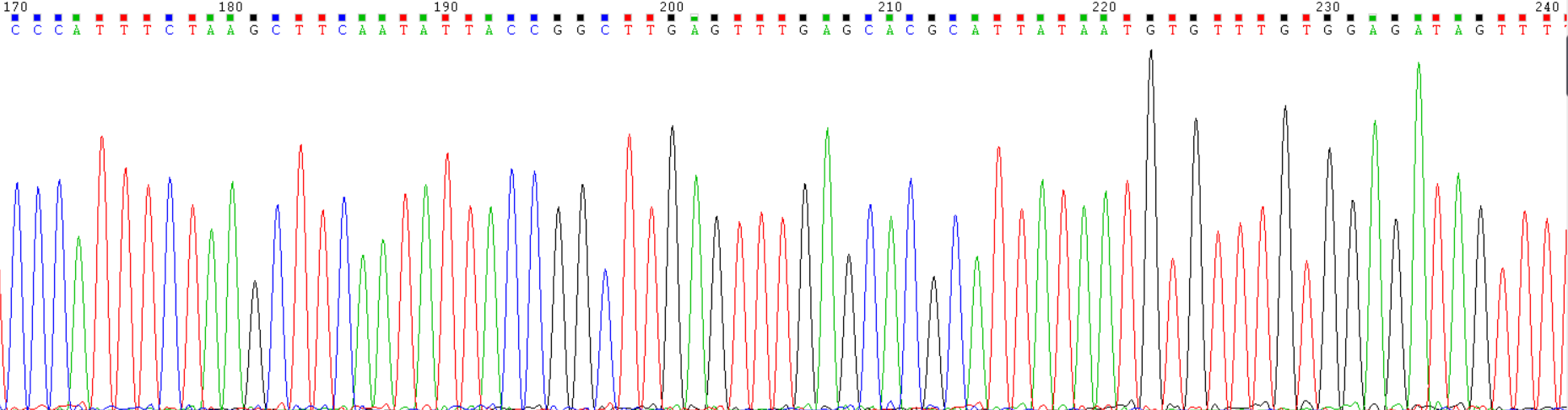


***Eomesb1***


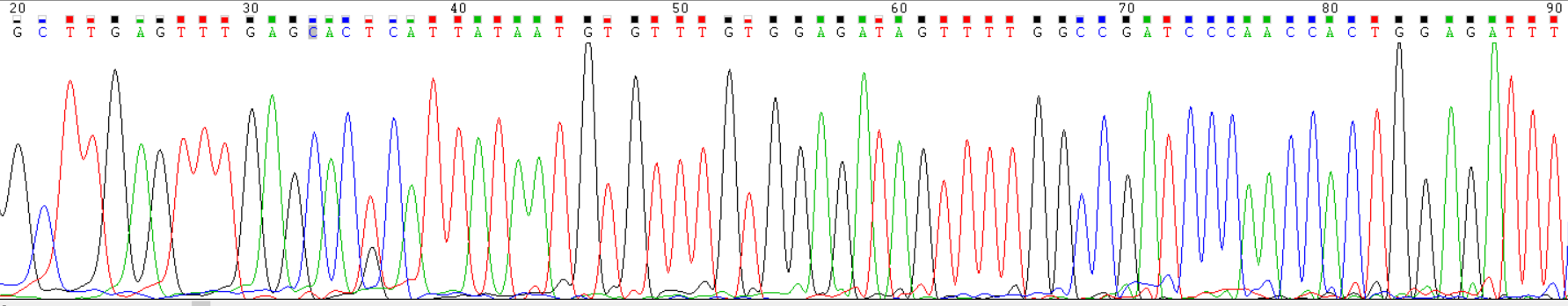

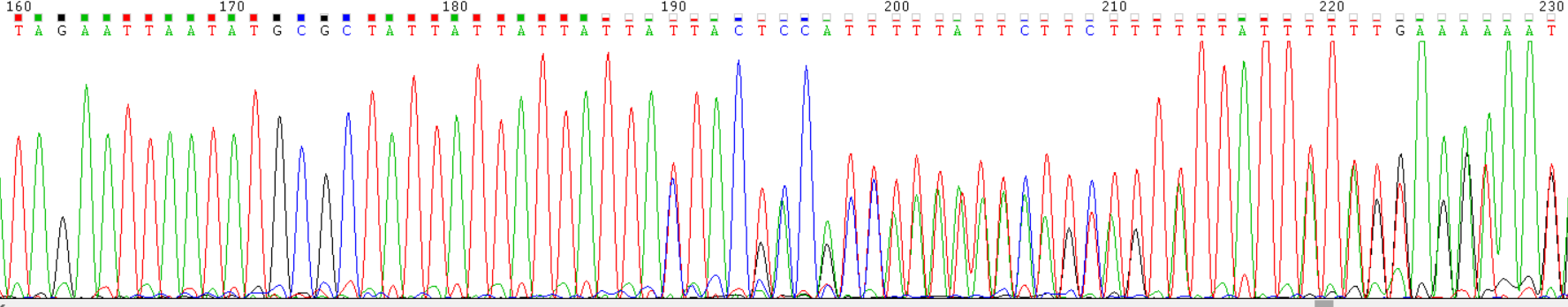

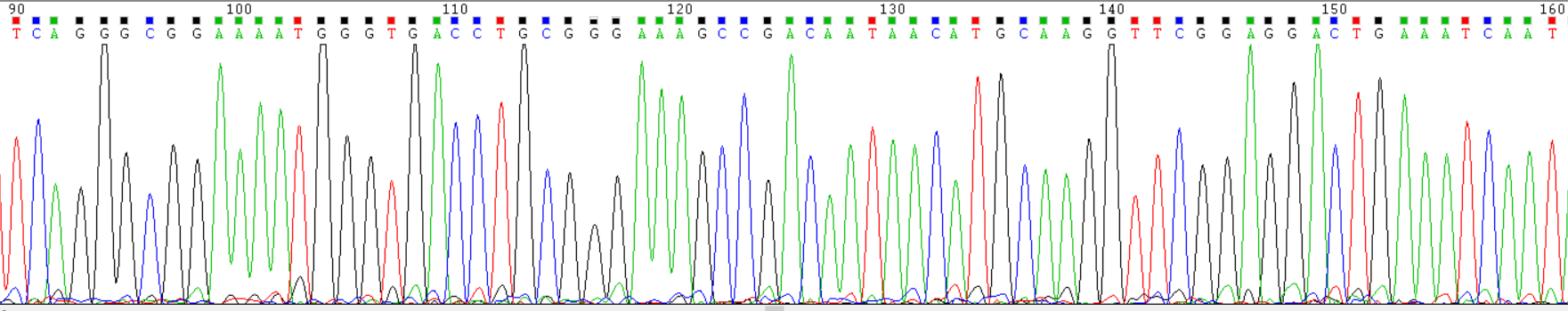


***Eomesb2***

**Larvae 02**

***eomesb2***

**TA clone sequencing result:** **Five clones with indels among the ten examined clones**

**Reference:** AGCACTCATTATAATGTGTTTGTGGAGATAGTTTTGGCCGATCCCAACCACTGGAGATTTCAGGGC

GGAAAATGGGTGACCTGCGGGAAAGCCGACAATAACATGCAAGGTTCGGAGGACTGAAATCAAT

AGAATTAATATGCGCTATTATTATTATTACTACGCCATTTAAATTCAGTTTTATTATTTTAGGAAATAAG

B2-2-03: AGCACTCATTATAATGTGTTTGTGGAGATAGTTTTGGCCGATCCCAACCACTGGAGATTTCAGGGC

GGAAAATGGGTGACCTGCGGGAAAGCCGACAATAACATGCAAGGTTCGGAGGACTGAAATCAAT

AGAATTAATATGCGC---TATTATTATTATTATTACTACGCCATTTAAATTCAGTTTTATTATTTTAGGA (-3 bp)

b2-2-04: AGCACTCATTATAATGTGTTTGTGGAGATAGTTTTGGCCGATCCCAACCACTGGAGATTTCAGGGC

GGAAAATGGGTGACCTGCGGGAAAGCCGACAATAACATGCAAGGTTCGGAGGACTGAAATCAAT

AGAATTAATATGCGC---TATTATTATTATTATTACTACGCCATTTAAATTCAGTTTTATTATTTTAGGA (-3 bp)

b2-2-05: AGCACTCATTATAATGTGTTTGTGGAGATAGTTTTGGCCGATCCCAACCACTGGAGATTTCAGGGC

GGAAAATGGGTGACCTGCGGGAAAGCCGACAATAACATGCAAGGTTCGGAGGACTGAAATCAAT

AGAATTAATATGCGC---TATTATTATTATTATTACTACGCCATTTAAATTCAGTTTTATTATTTTAGGA (-3 bp)

b2-2-06: AGCACTCATTATAATGTGTTTGTGGAGATAGTTTTGGCCGATCCCAACCACTGGAGATTTCAGGGC

GGAAAATGGGTGACCTGCGGGAAAGCCGACAATAACATGCAAGGTTCGGAGGACTGAAATCAAT

AGAATTAATATGCGC---TATTATTATTATTATTACTACGCCATTTAAATTCAGTTTTATTATTTTAGGA (-3 bp)

b2-2-07: AGCACTCATTATAATGTGTTTGTGGAGATAGTTTTGGCCGATCCCAACCACTGGAGATTTCAGGGC

GGAAAATGGGTGACCTGCGGGAAAGCCGACAATAACATGCAAGGTTCGGAGGACTGAAATCAAT

AGAATTAATATGCGC---TATTATTATTATTATTACTACGCCATTTAAATTCAGTTTTATTATTTTAGGA (-3 bp)

**Larvae 03**

**PCR sequencing result**


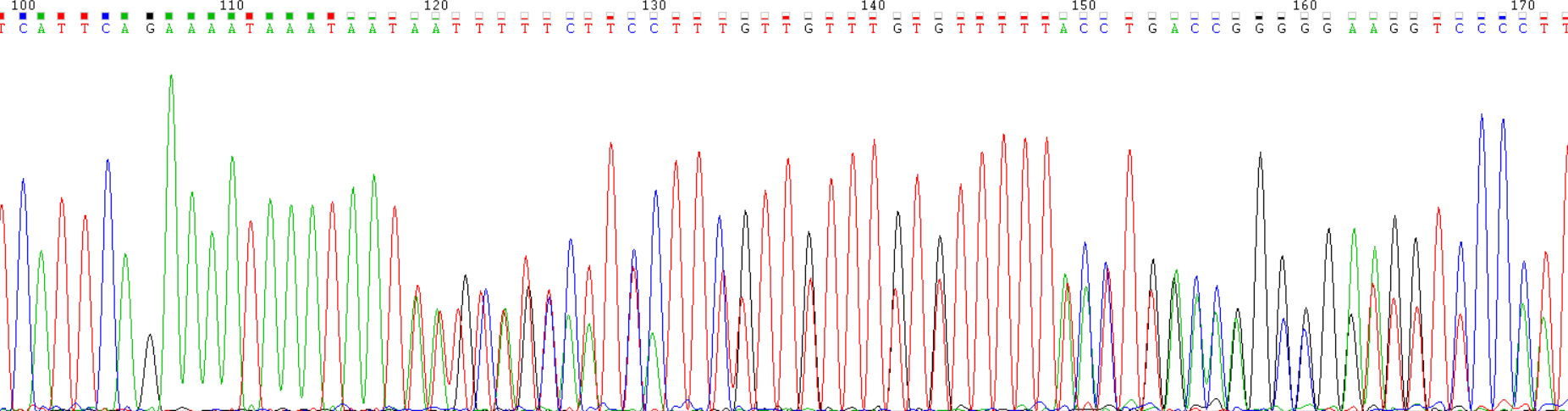

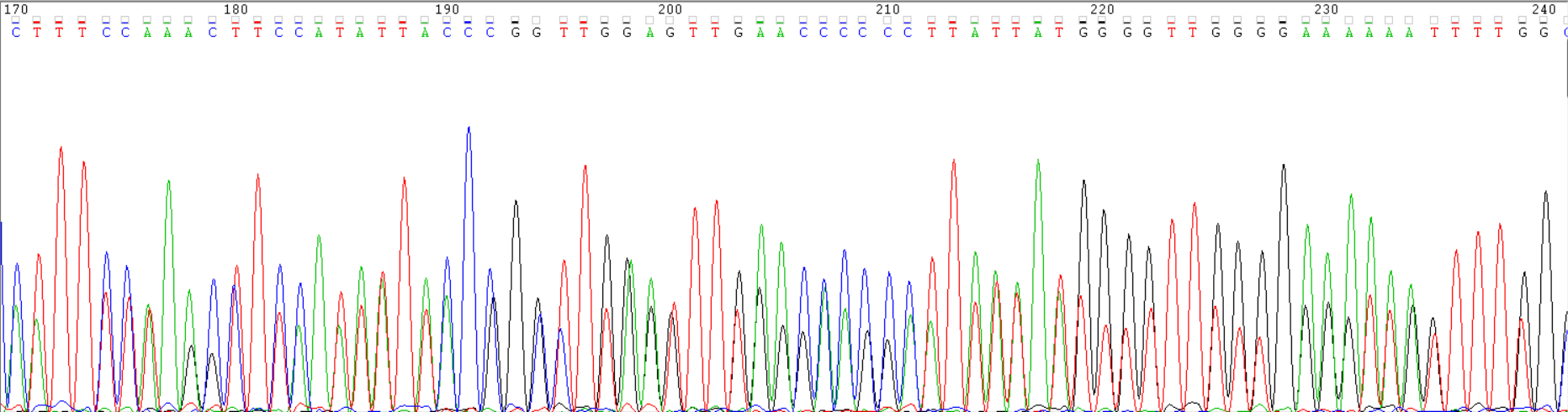


***Eomesb1***

***Eomesb2***


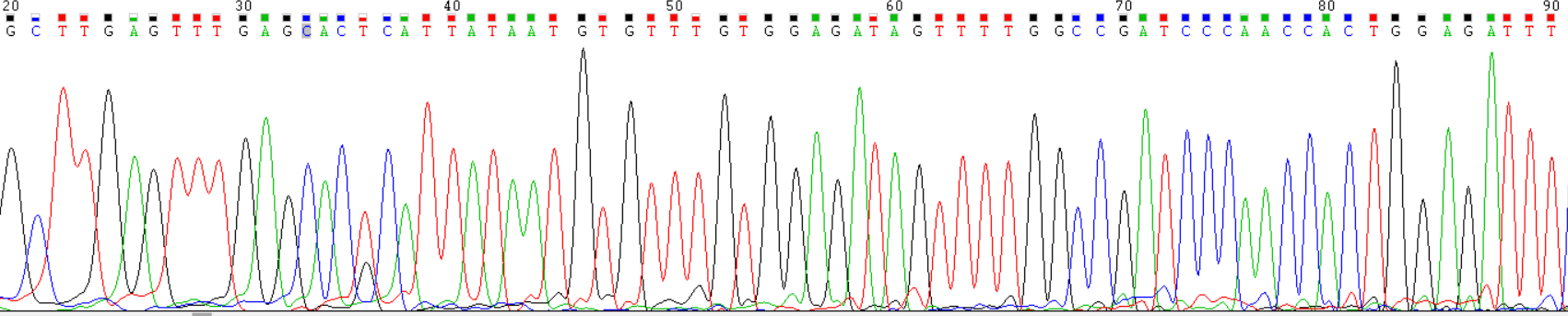

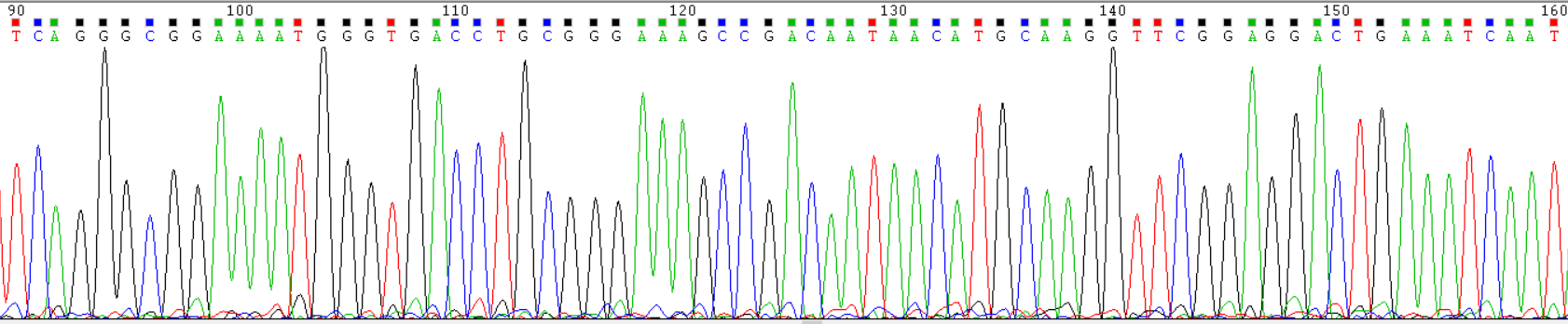

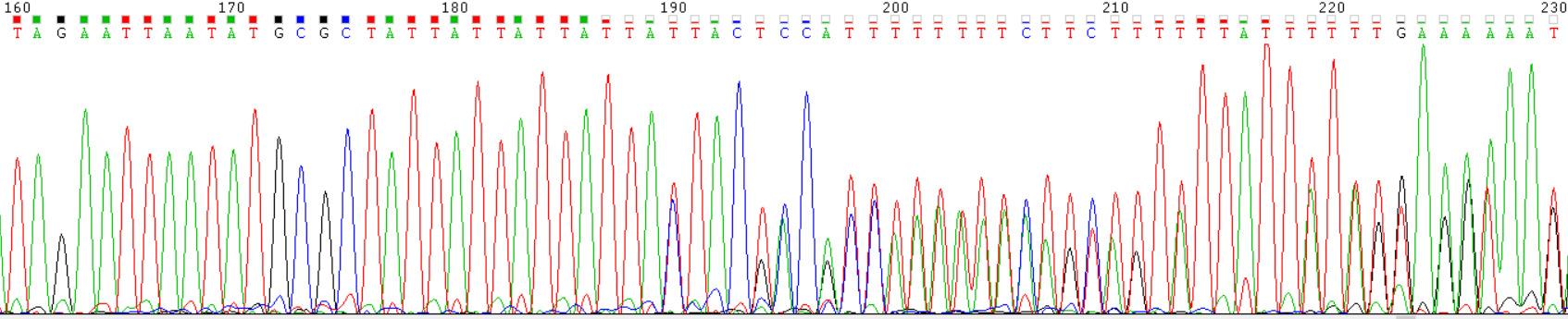


**Larvae 03**

***eomesb1***

**TA clone sequencing result:** **Ten clones with indels among the ten examined clones**

**Reference:** AAAATAAATAATAATTTGCATTCATTCTTTGTTTTTGTTTTTTACTTGACAGGCGGATGTTCCCATT

TCTAAGCTTCAATATTACCGGCTTGAGTTTGAGCACGCATTATAATGTGTTTGTGGAGATAGTTTTGG

B1-3-01: ATAAATAATAATTTGCATTCATTCTTTGTTTTTGT-TTTTTACTT----GACAGGCGGATGTTCCCATT

TCTAAGCTTCAATATTACCGGCTTGAGTTTGAGCACGCATTATAATGTGTTTGTGGAGATAGTTTTGG (-4 bp)

B1-3-02: ATAAATAATAATTTGCATTCATTCTTTGTTTTTGT-TTTTTACTT----GACAGGCGGATGTTCCCATT

TCTAAGCTTCAATATTACCGGCTTGAGTTTGAGCACGCATTATAATGTGTTTGTGGAGATAGTTTTGG (-4 bp)

B1-3-03: ATAAATAATAATTTGCATTCATTCTTTGTTTTTGT-TTTTTACTT----GACAGGCGGATGTTCCCATT

TCTAAGCTTCAATATTACCGGCTTGAGTTTGAGCACGCATTATAATGTGTTTGTGGAGATAGTTTTGG (-4 bp)

B1-3-04: ATAAATAATAATTTGCATTCATTCTTTGTTTTTGT-TTTTTACTT----GACAGGCGGATGTTCCCATT

TCTAAGCTTCAATATTACCGGCTTGAGTTTGAGCACGCATTATAATGTGTTTGTGGAGATAGTTTTGG (-4 bp)

B1-3-05: ATA---AATAATTTGCATTCATTCTTTGTTTTTTGTTTTTTTACTTGACAGGCGGATGTTCCCATTTC

TAAGCTTCAATATTACCGGCTTGAGTTTGAGCACGCATTATAATGTGTTTGTGGAGATAGTTTTGG (-1 bp)

B1-3-06: ATAAATAATAATTTGCATTCATTCTTTGTTTTTGT-TTTTTACTT----GACAGGCGGATGTTCCCATT

TCTAAGCTTCAATATTACCGGCTTGAGTTTGAGCACGCATTATAATGTGTTTGTGGAGATAGTTTTGG (-4 bp)

B1-3-07: ATAAATAATAATTTGCATTCATTCTTTGTTTTTGT-TTTTTACTT----GACAGGCGGATGTTCCCATT

TCTAAGCTTCAATATTACCGGCTTGAGTTTGAGCACGCATTATAATGTGTTTGTGGAGATAGTTTTGG (-4 bp)

B1-3-08: ATAAATAATAATTTGCATTCATTCTTTGTTTTTGT-TTTTTACTT----GACAGGCGGATGTTCCCATT

TCTAAGCTTCAATATTACCGGCTTGAGTTTGAGCACGCATTATAATGTGTTTGTGGAGATAGTTTTGG (-4 bp)

B1-3-09: ATAAATAATAATTTGCATTCATTCTTTGTTTTTGT-TTTTTACTT----GACAGGCGGATGTTCCCATT

TCTAAGCTTCAATATTACCGGCTTGAGTTTGAGCACGCATTATAATGTGTTTGTGGAGATAGTTTTGG (-4 bp)

B1-3-10: ATA---AATAATTTGCATTCATTCTTTGTTTTTTGTTTTTTTACTTGACAGGCGGATGTTCCCATTTC

TAAGCTTCAATATTACCGGCTTGAGTTTGAGCACGCATTATAATGTGTTTGTGGAGATAGTTTTGG (-1 bp)

**Larvae 04**

**PCR sequencing result**


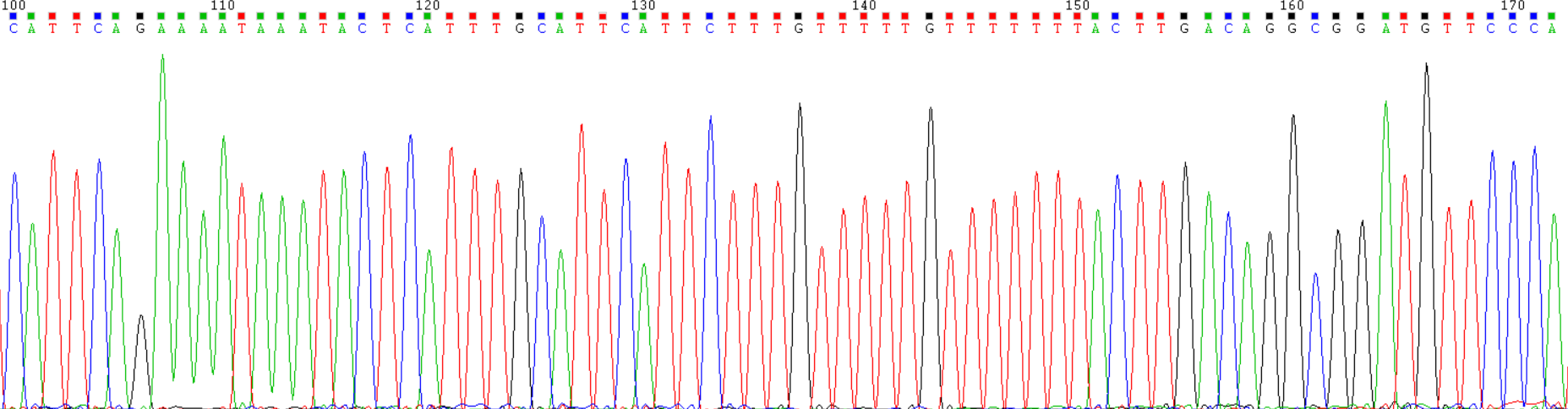

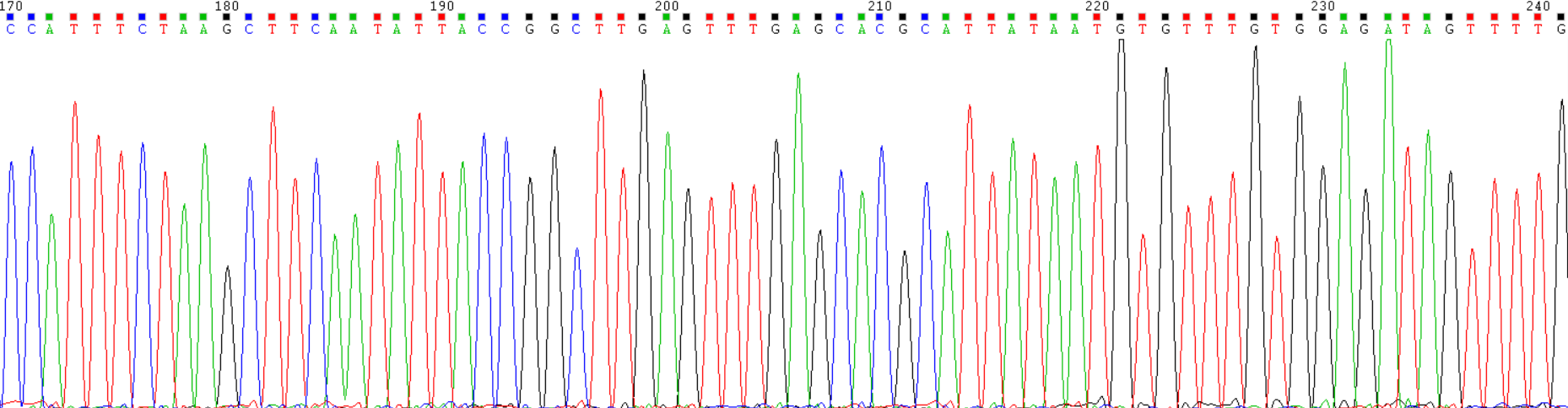


***Eomesb1***

***Eomesb2***


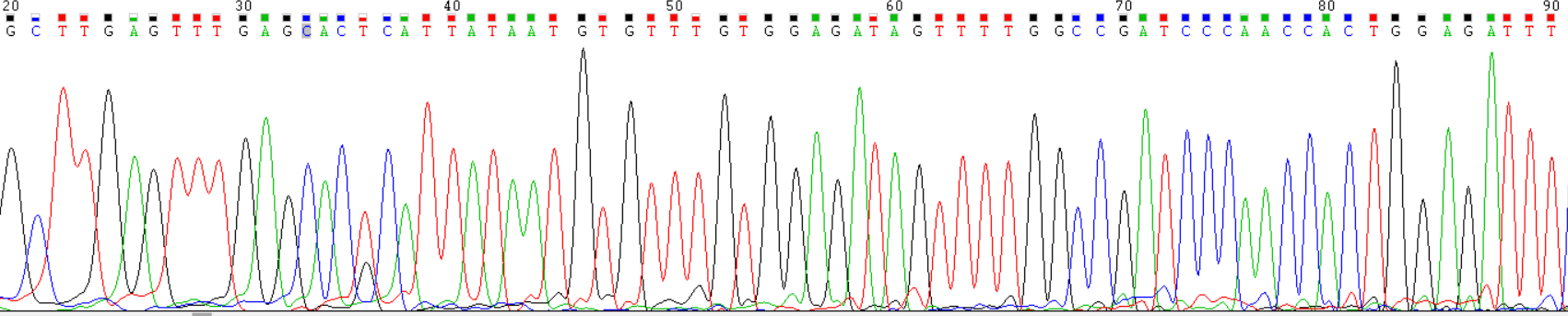

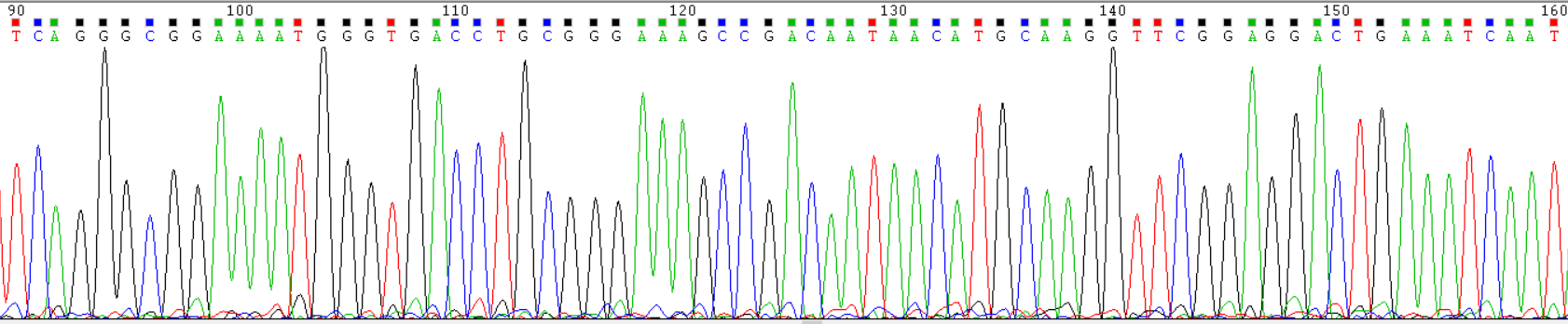

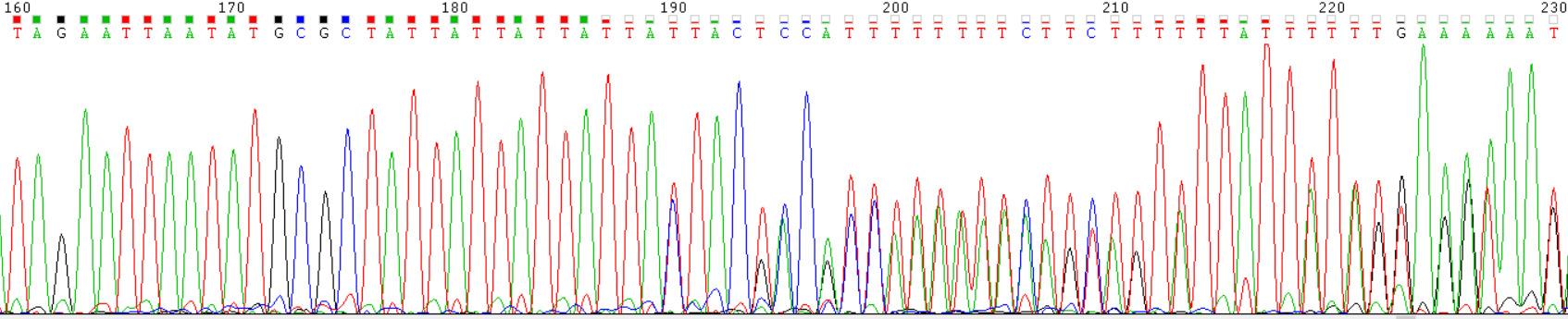


**Larvae 04**

***eomesb2***

**TA clone sequencing result :Two clones with indels among the ten examined clones**

**Reference:** AGCACTCATTATAATGTGTTTGTGGAGATAGTTTTGGCCGATCCCAACCACTGGAGATTTCAGGGC

GGAAAATGGGTGACCTGCGGGAAAGCCGACAATAACATGCAAGGTTCGGAGGACTGAAATCAAT

AGAATTAATATGCGCTATTATTATTATTACTACGCCATTTAAATTCAGTTTTATTATTTTAGGAAATAAG

B2-4-03: AGCACTCATTATAATGTGTTTGTGGAGATAGTTTTGGCCGATCCCAACCACTGGAGATTTCAGGGC

GGAAAATGGGTGACCTGCGGGAAAGCCGACAATAACATGCAAGGTTCGGAGGACTGAAATCAAT

AGAATTAATATGCGCTATTATTATTATTATTACTACGCCATTTAAATTCAGTTTTATTATTTTAGGAAAT (+3 bp)

B2-4-04: AGCACTCATTATAATGTGTTTGTGGAGATAGTTTTGGCCGATCCCAACCACTGGAGATTTCAGGGC

GGAAAATGGGTGACCTGCGGGAAAGCCGACAATAACATGCAAGGTTCGGAGGACTGAAATCAAT

AGAATTAATATGCGCTATTATTATTATTATTACTACGCCATTTAAATTCAGTTTTATTATTTTAGGAAAT (+3 bp)

**Larvae 05**

**PCR sequencing result**

***Eomesb2***


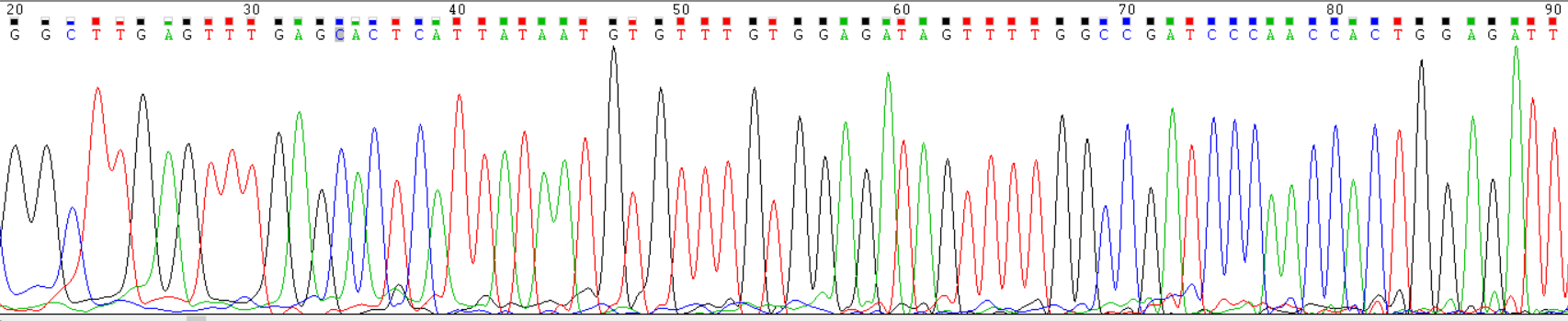

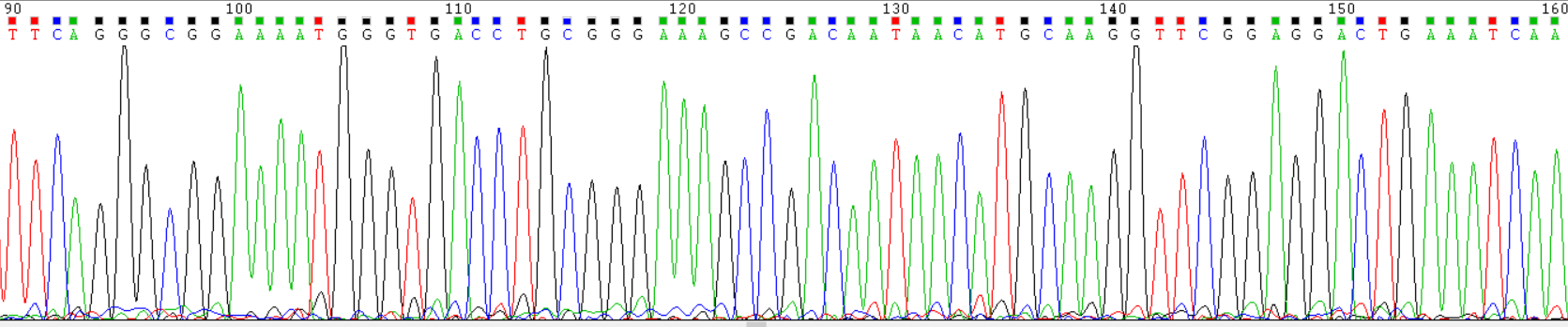

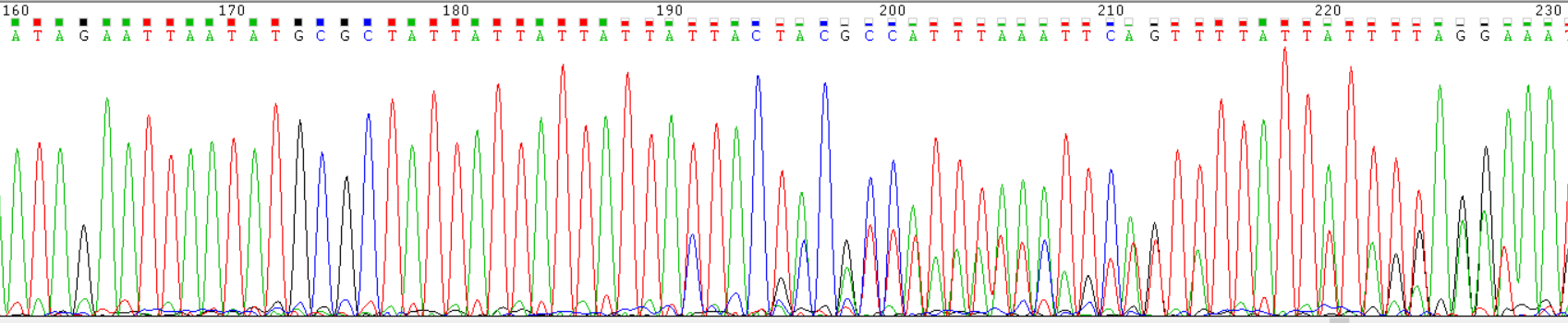

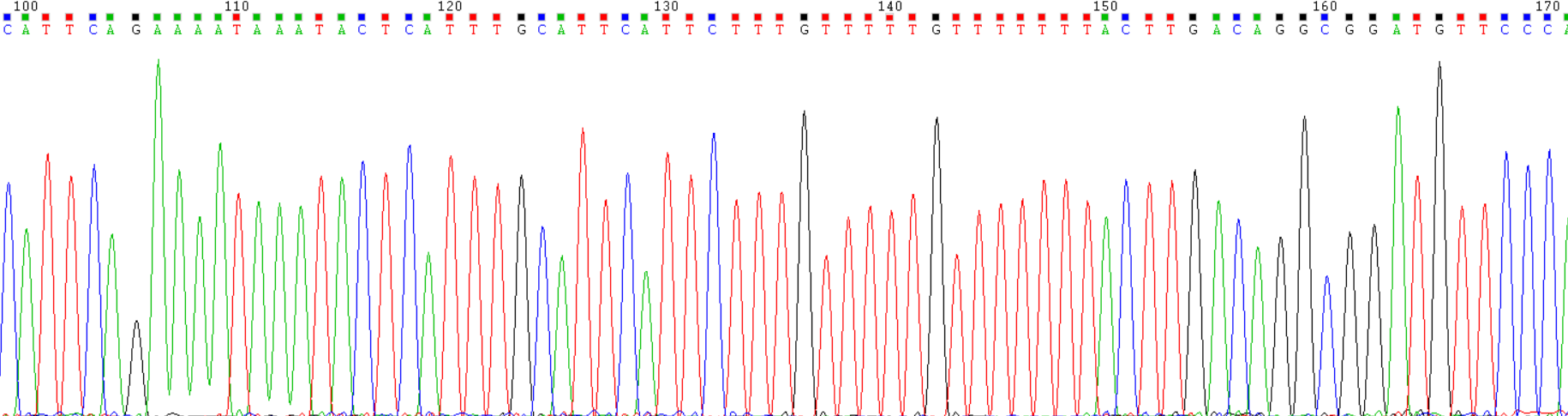

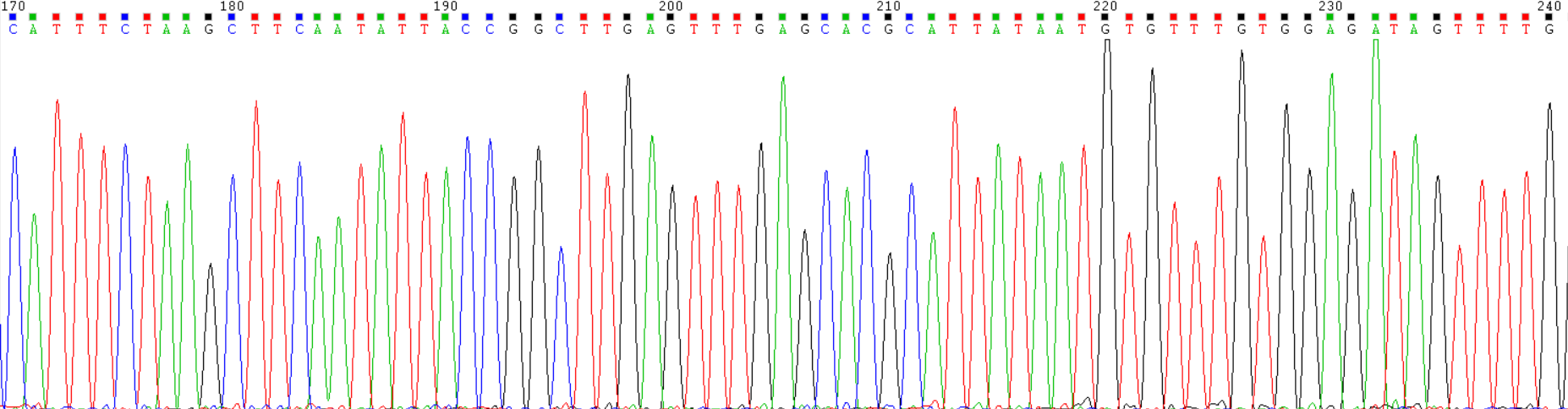


***Eomesb1***

**Larvae 06**

**PCR sequencing result**


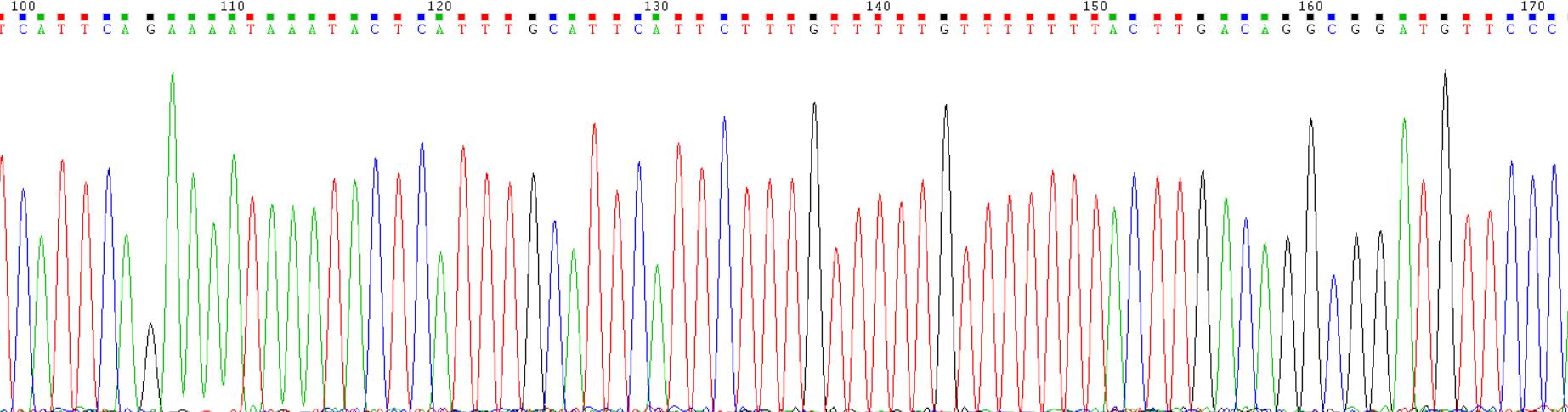

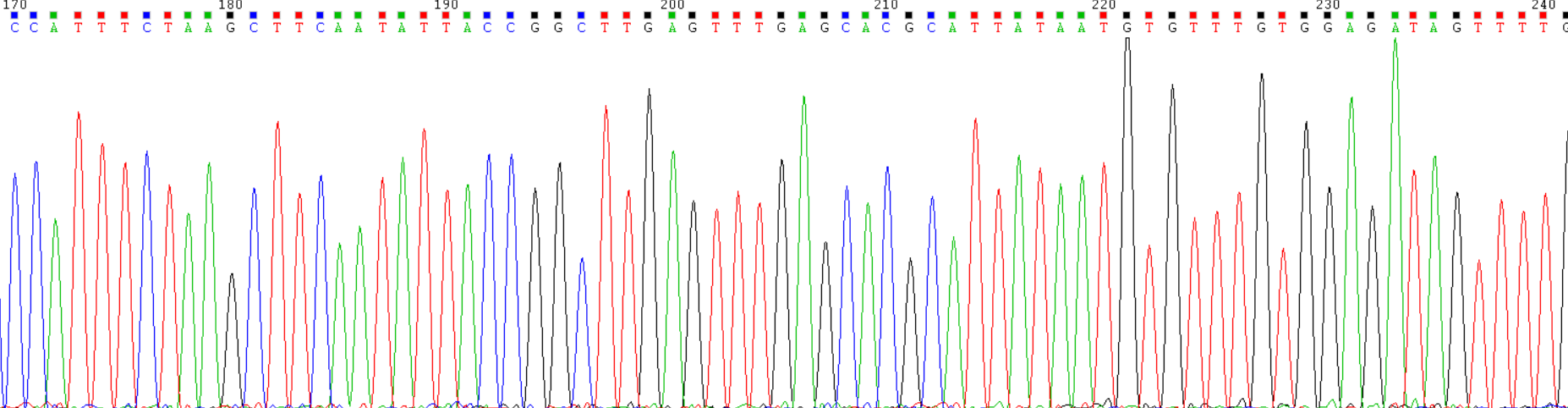


***Eomesb1***

***Eomesb2***


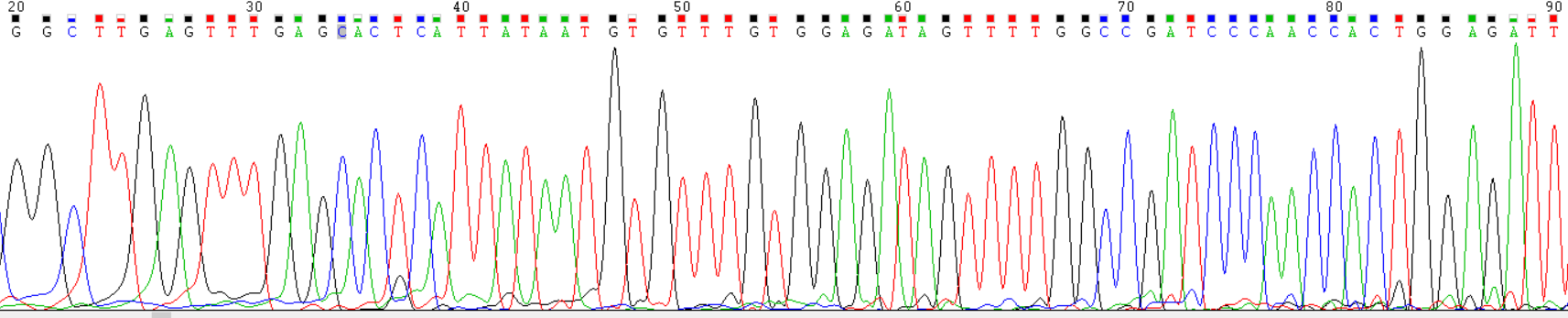

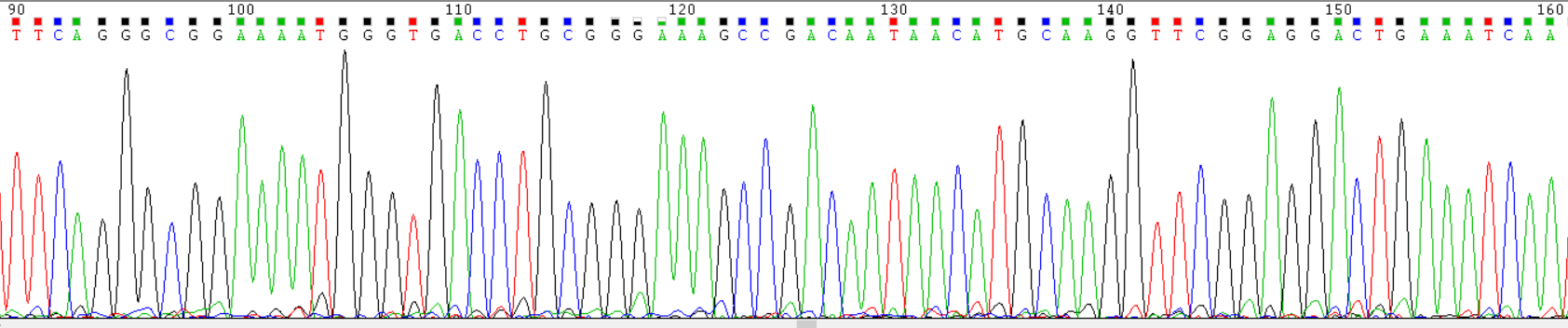

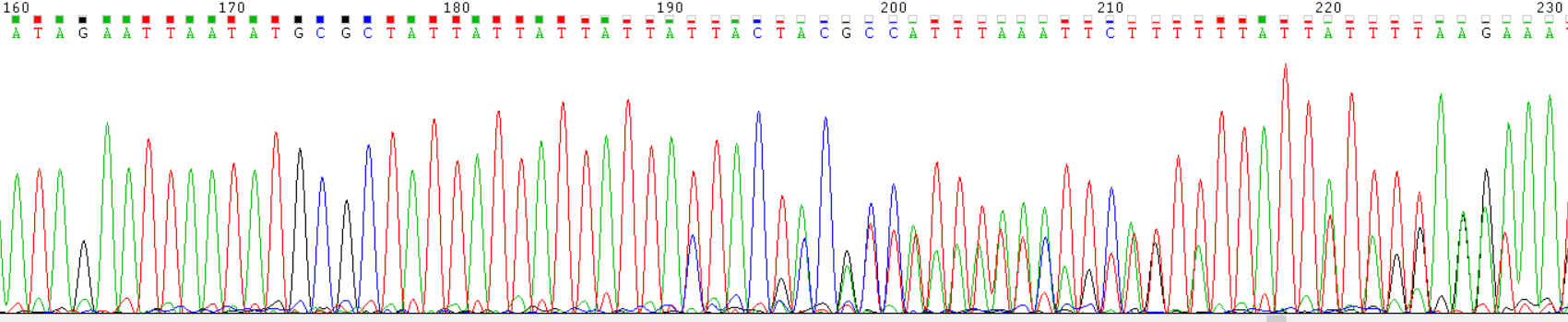


**Larvae 06**

***eomesb2***

**TA clone sequencing result:** **Three clones with indels among the ten examined clones**

**Reference:** AGCACTCATTATAATGTGTTTGTGGAGATAGTTTTGGCCGATCCCAACCACTGGAGATTTCAGGGC

GGAAAATGGGTGACCTGCGGGAAAGCCGACAATAACATGCAAGGTTCGGAGGACTGAAATCAAT

AGAATTAATATGCGCTATTATTATTATTACTACGCCATTTAAATTCAGTTTTATTATTTTAGGAAATAAG

B2-6-02: AGCACTCATTATAATGTGTTTGTGGAGATAGTTTTGGCCGATCCCAACCACTGGAGATTTCAGGGC

GGAAAATGGGTGACCTGCGGGAAAGCCGACAATAACATGCAAGGTTCGGAGGACTGAAATCAAT

AGAATTAATATGCGCTATTATTATTATTATTACTACGCCATTTAAATTCAGTTTTATTATTTTAGGAAAT (+3 bp)

B2-6-07: AGCACTCATTATAATGTGTTTGTGGAGATAGTTTTGGCCGATCCCAACCACTGGAGATTTCAGGGC

GGAAAATGGGTGACCTGCGGGAAAGCCGACAATAACATGCAAGGTTCGGAGGACTGAAATCAAT

AGAATTAATATGCGCTATTATTATTATTATTACTACGCCATTTAAATTCAGTTTTATTATTTTAGGAAAT (+3 bp)

B2-6-12: AGCACTCATTATAATGTGTTTGTGGAGATAGTTTTGGCCGATCCCAACCACTGGAGATTTCAGGGC

GGAAAATGGGTGACCTGCGGGAAAGCCGACAATAACATGCAAGGTTCGGAGGACTGAAATCAAT

AGAATTAATATGCGCTATTATTATTATTATTACTACGCCATTTAAATTCAGTTTTATTATTTTAGGAAAT (+3 bp)

**Larvae 07**

**PCR sequencing result**


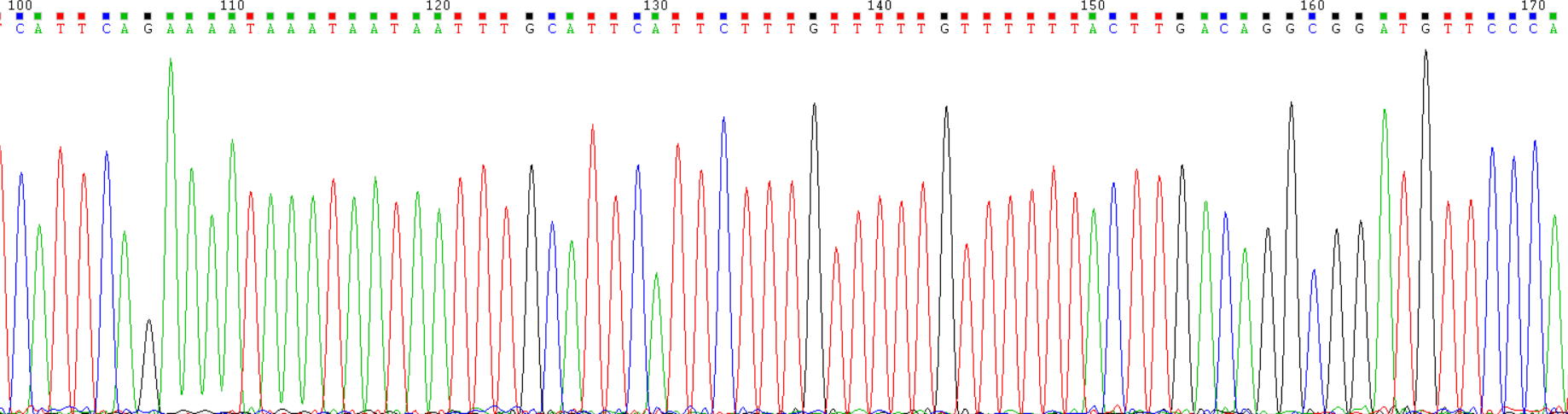

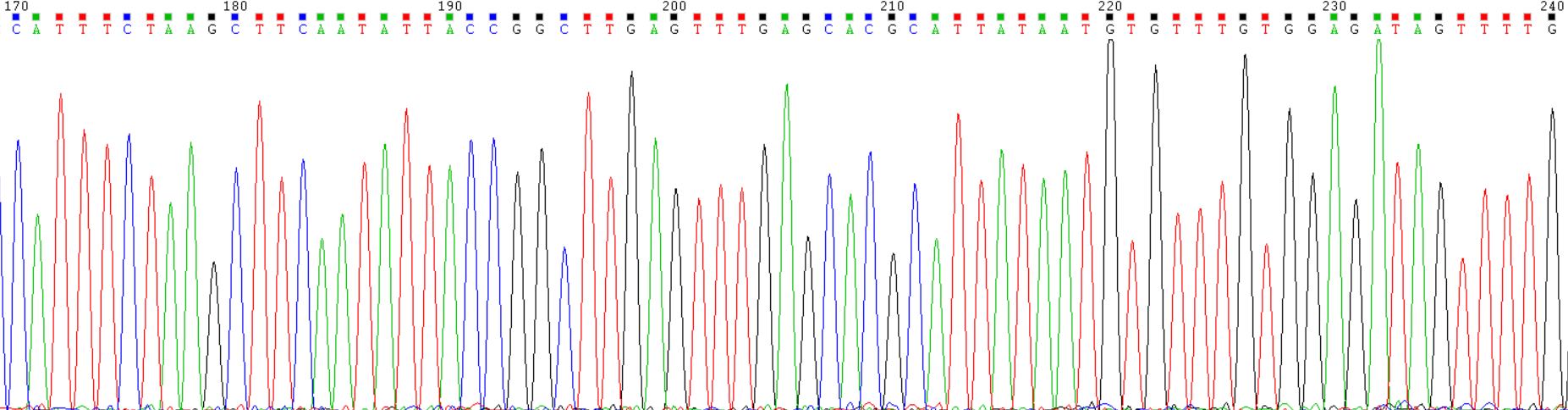


***Eomesb1***

***Eomesb2***


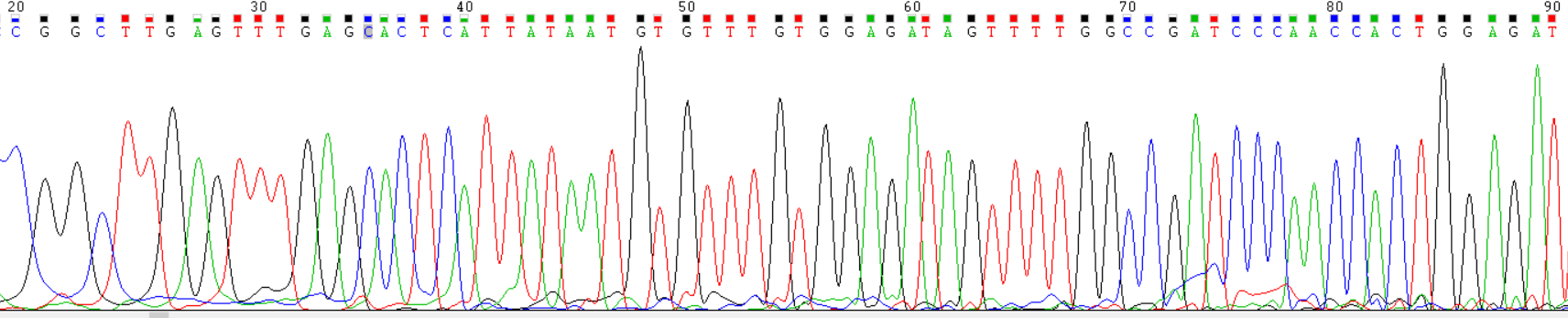

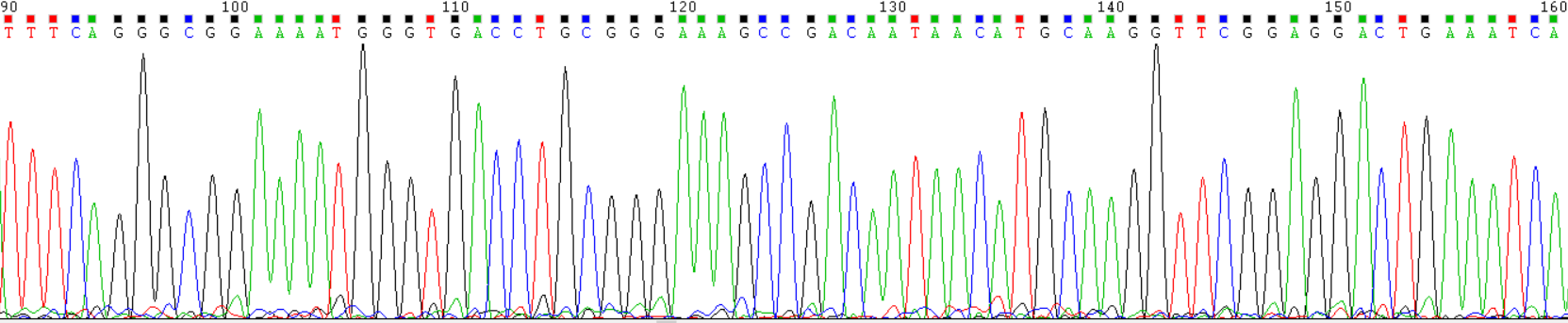

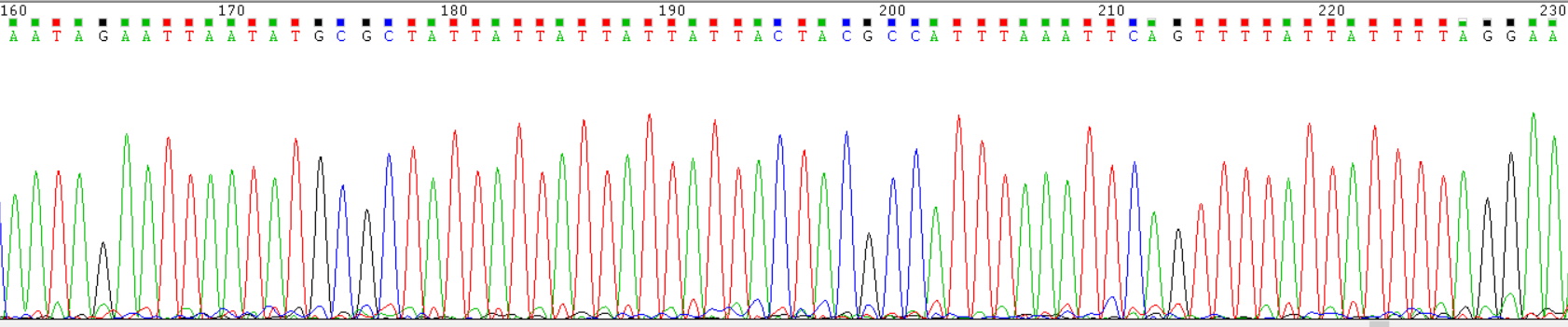


**Larvae 08**

**PCR sequencing result**


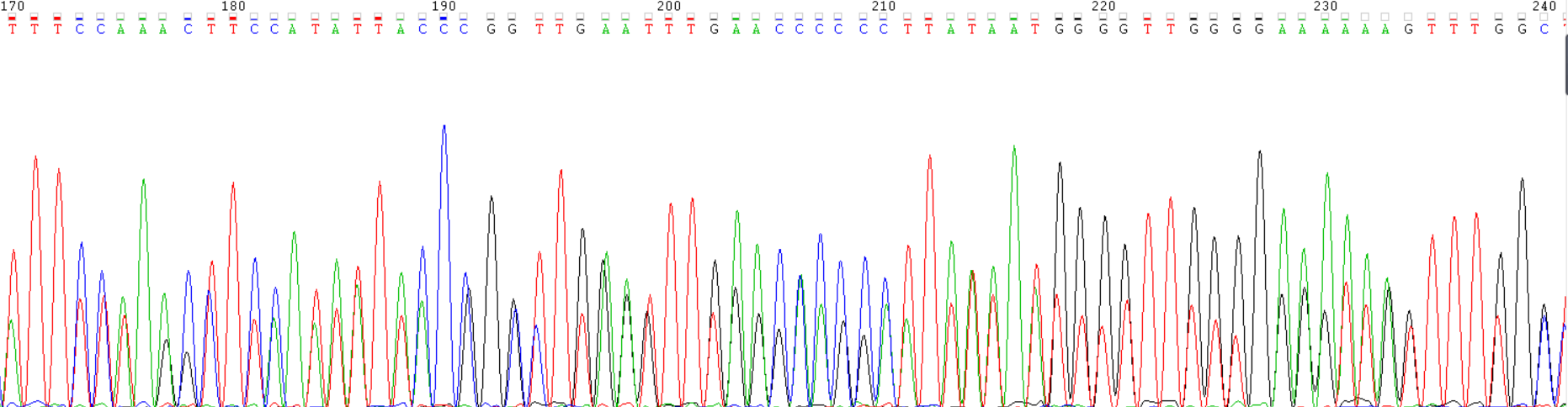

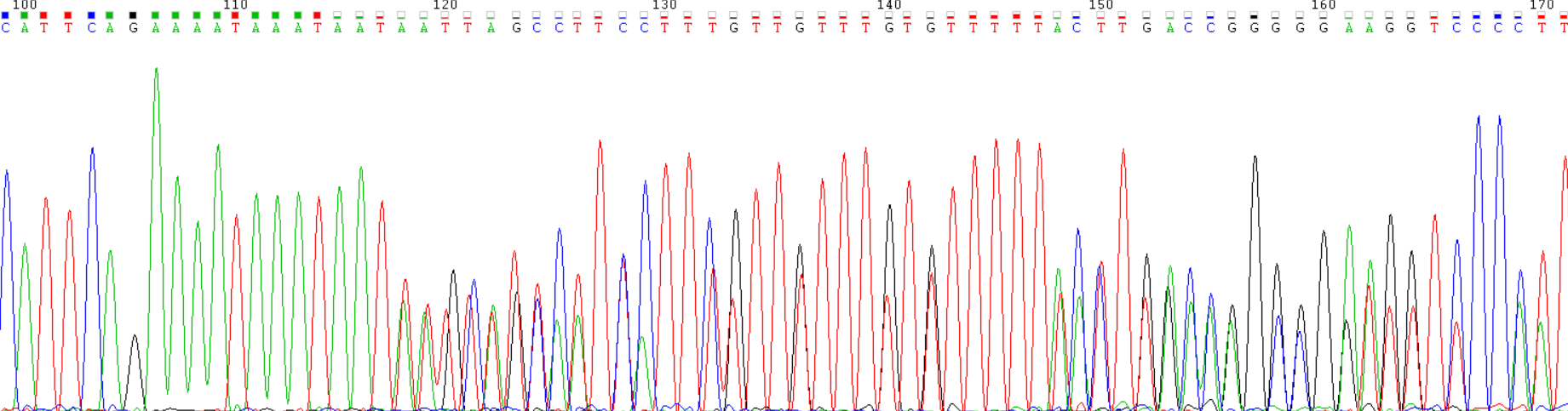


***Eomesb1***

***Eomesb2***


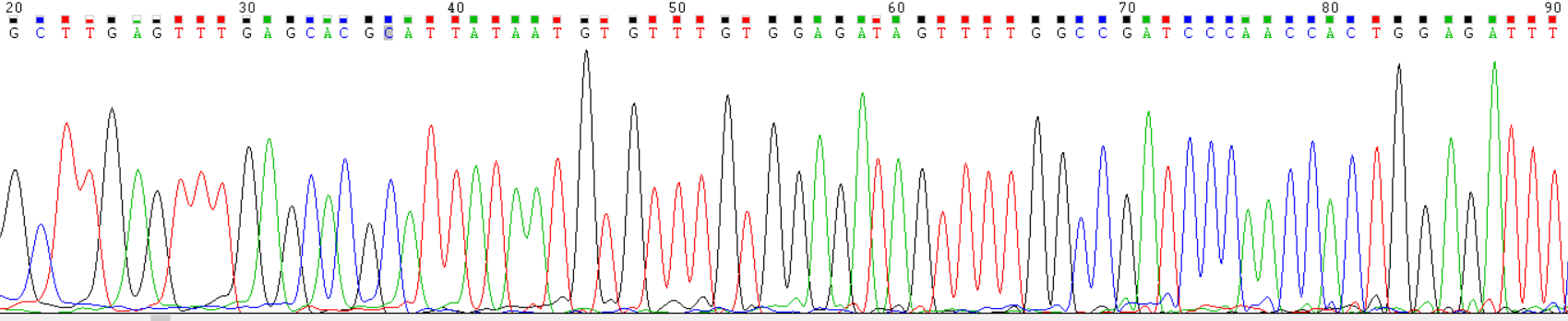

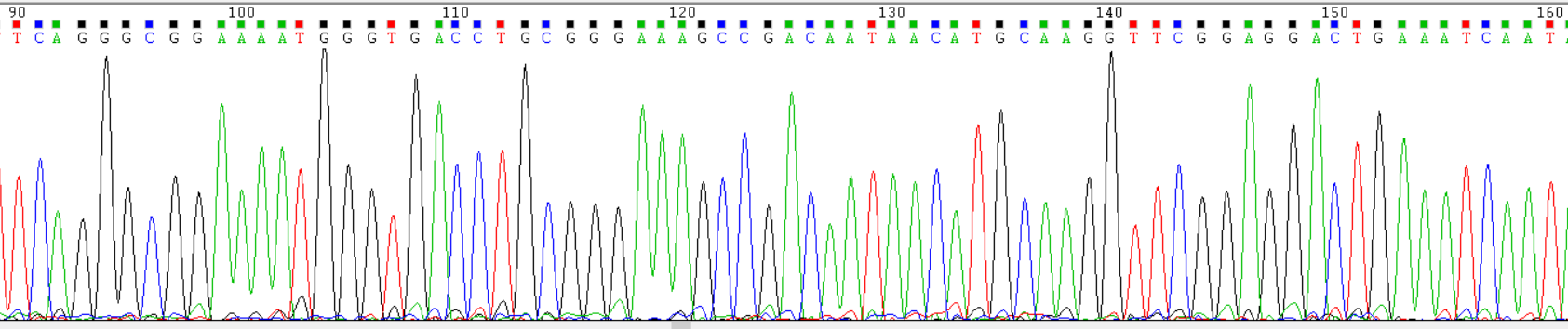

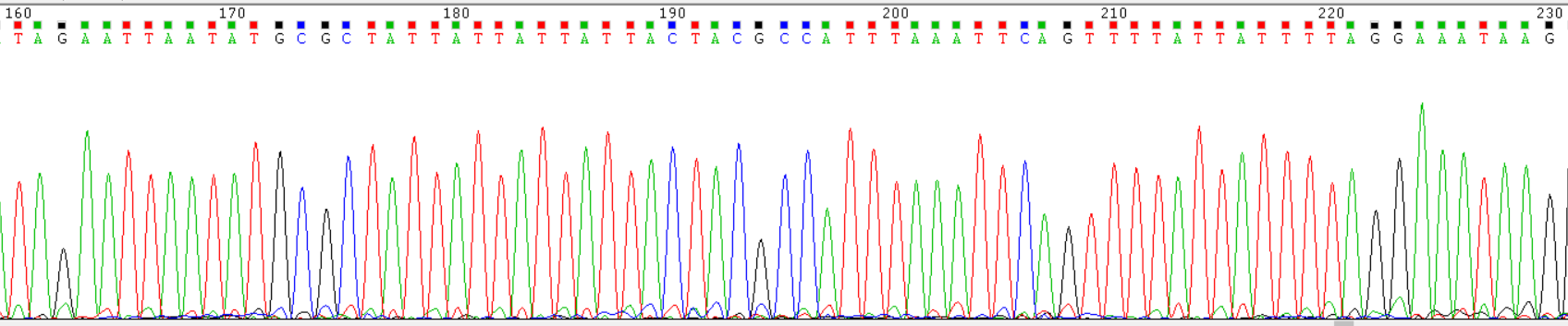


**Larvae 08**

***eomesb1* TA**

**TA clone sequencing result:** **Five clones with indels among the ten examined clones**

**Reference:** AAAATAAATAATAATTTGCATTCATTCTTTGTTTTTGTTTTTTACTTGACAGGCGGATGTTCCCATTTC

TAAGCTTCAATATTACCGGCTTGAGTTTGAGCACGCATTATAATGTGTTTGTGGAGATAGTTTTGG

B1-8-06: ATA---AATAATTTGCATTCATTCTTTGTTTTTTGTTTTTTTACTTGACAGGCGGATGTTCCCATTTC

TAAGCTTCAATATTACCGGCTTGAGTTTGAGCACGCATTATAATGTGTTTGTGGAGATAGTTTTGG (-1 bp)

B1-8-07: ATA---AATAATTTGCATTCATTCTTTGTTTTTTGTTTTTTTACTTGACAGGCGGATGTTCCCATTTC

TAAGCTTCAATATTACCGGCTTGAGTTTGAGCACGCATTATAATGTGTTTGTGGAGATAGTTTTGG (-1 bp)

B1-8-13: ATA---AATAATTTGCATTCATTCTTTGTTTTTTGTTTTTTTACTTGACAGGCGGATGTTCCCATTTC

TAAGCTTCAATATTACCGGCTTGAGTTTGAGCACGCATTATAATGTGTTTGTGGAGATAGTTTTGG (-1 bp)

B1-8-14: ATA---AATAATTTGCATTCATTCTTTGTTTTTTGTTTTTTTACTTGACAGGCGGATGTTCCCATTTC

TAAGCTTCAATATTACCGGCTTGAGTTTGAGCACGCATTATAATGTGTTTGTGGAGATAGTTTTGG (-1 bp)

B1-8-15: ATA---AATAATTTGCATTCATTCTTTGTTTTTTGTTTTTTTACTTGACAGGCGGATGTTCCCATTTC

TAAGCTTCAATATTACCGGCTTGAGTTTGAGCACGCATTATAATGTGTTTGTGGAGATAGTTTTGG (-1 bp)

**Larvae 09**

**PCR sequencing result**

***Eomesb2***


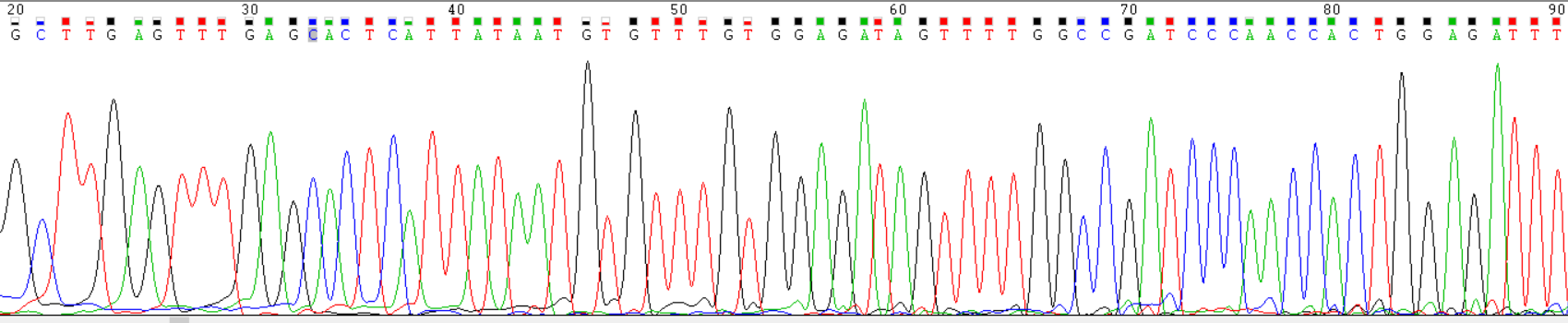

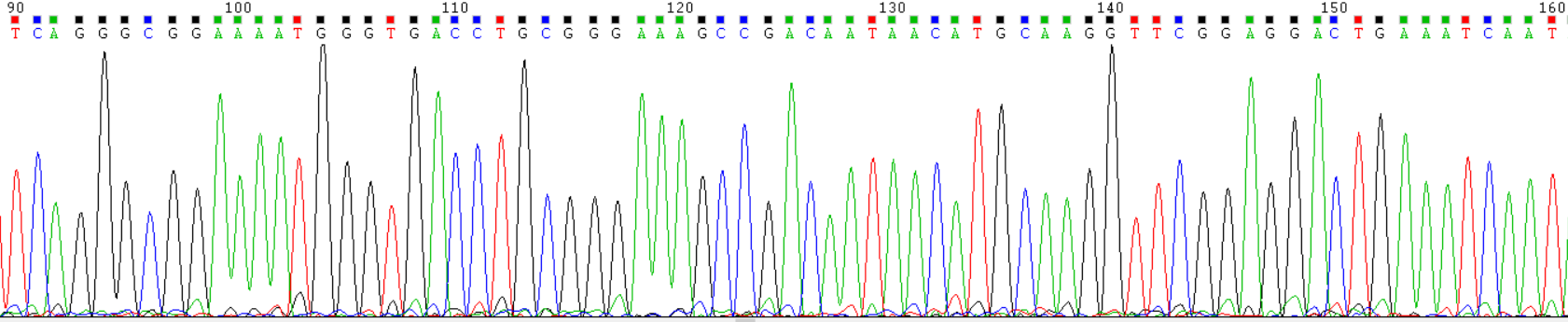

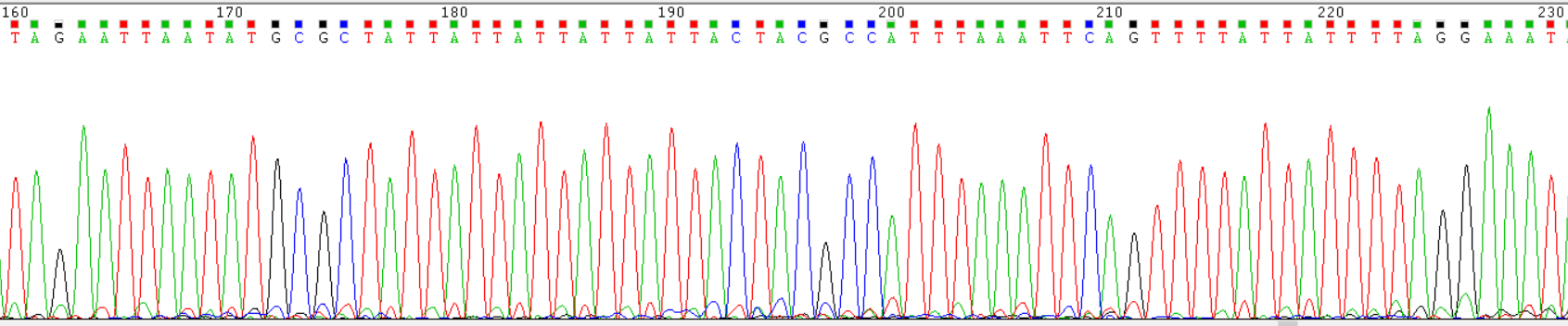

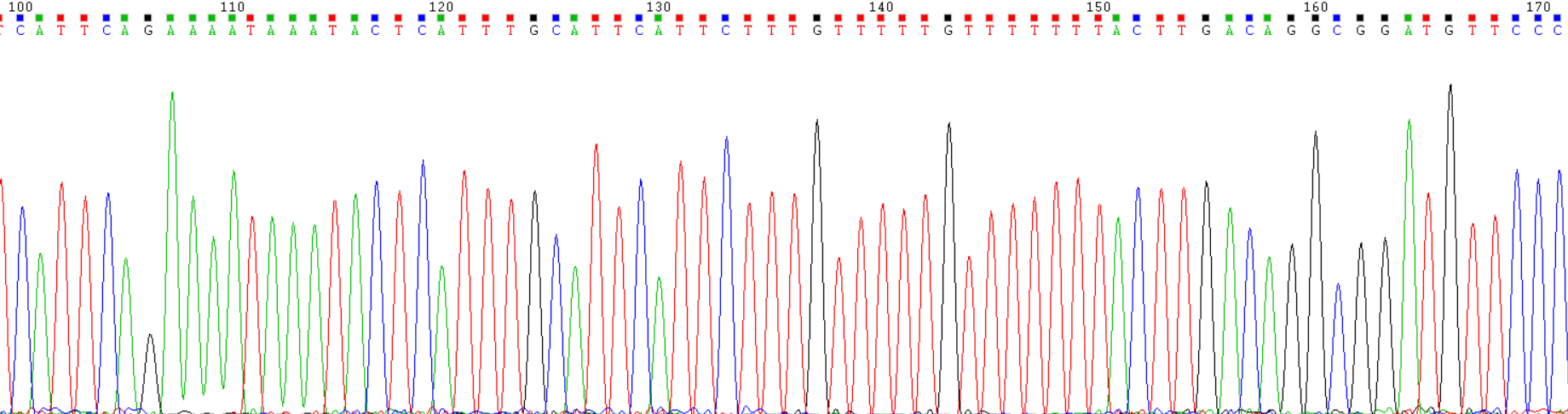

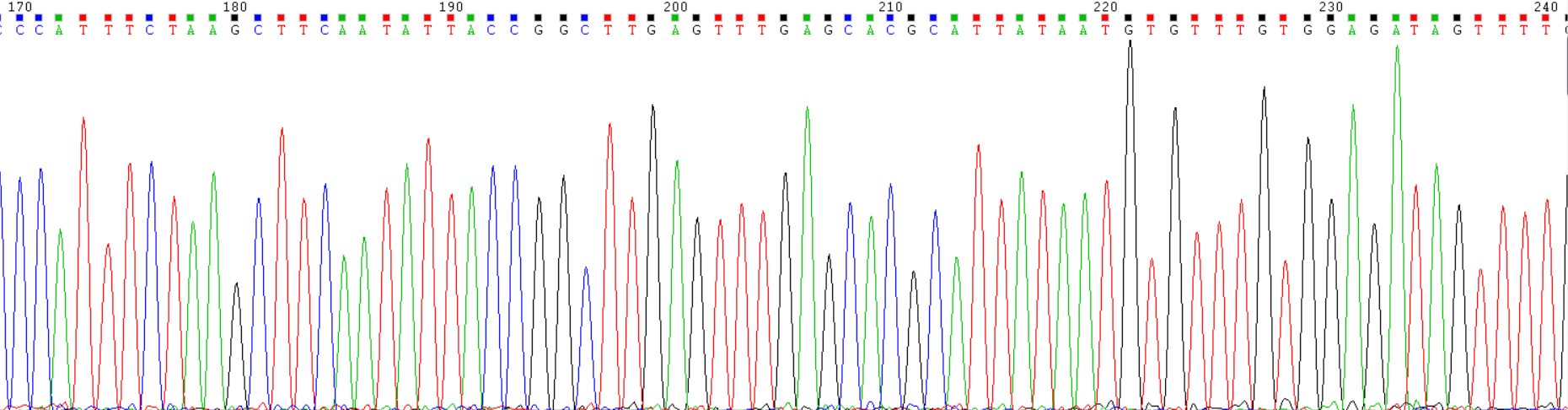


***Eomesb1***

**Larvae 10**

**PCR sequencing result**


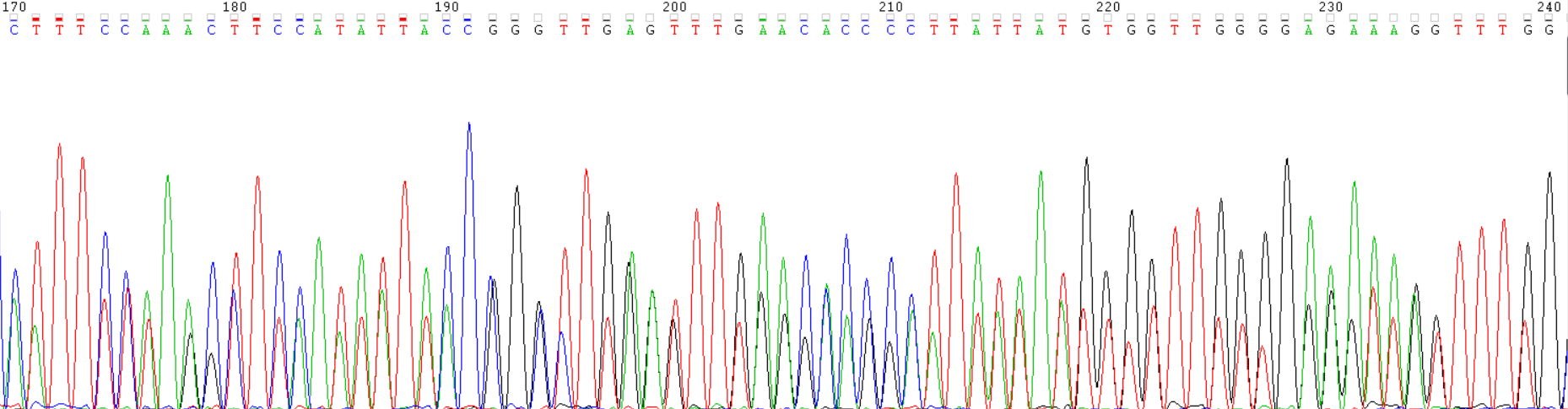

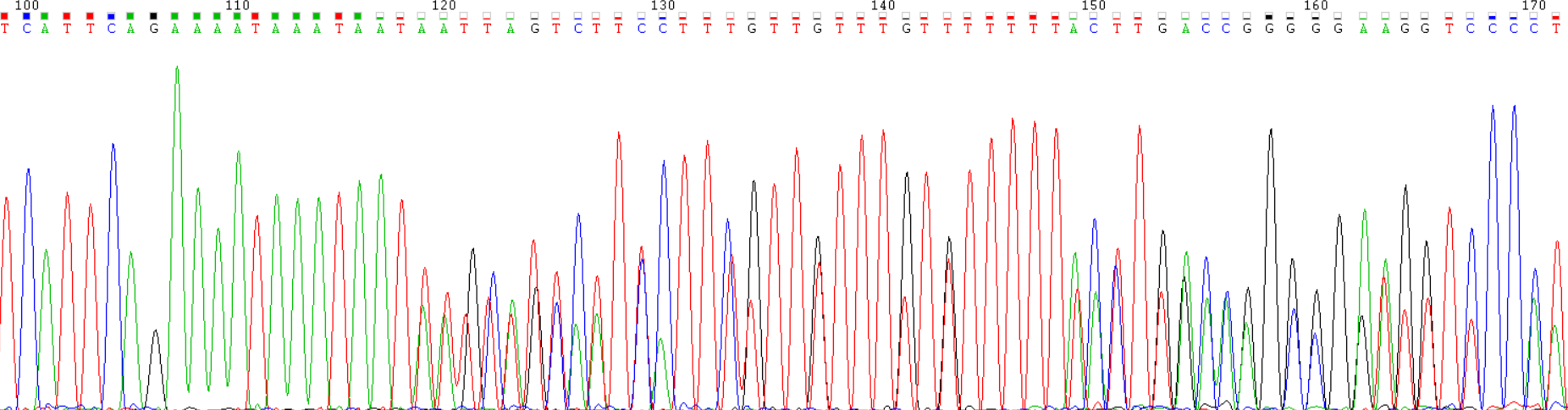


***Eomesb1***

***Eomesb2***


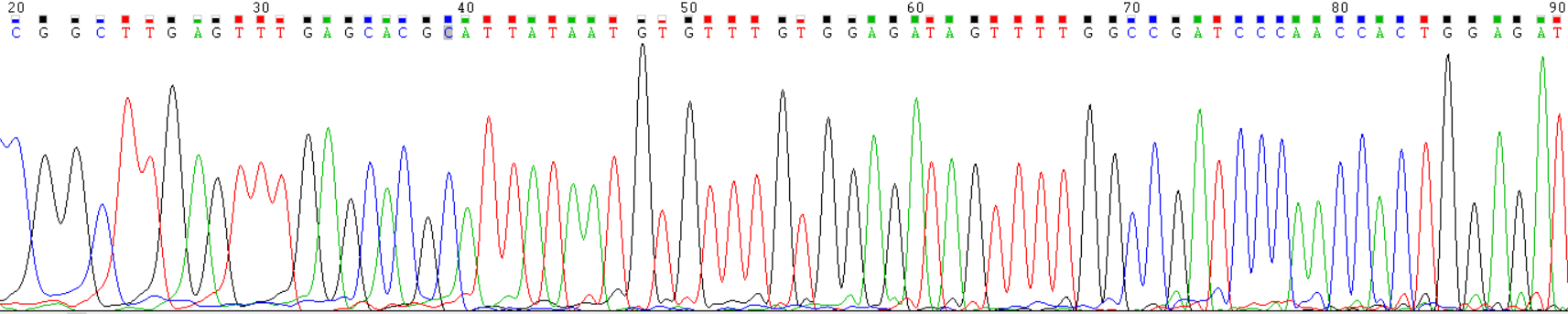

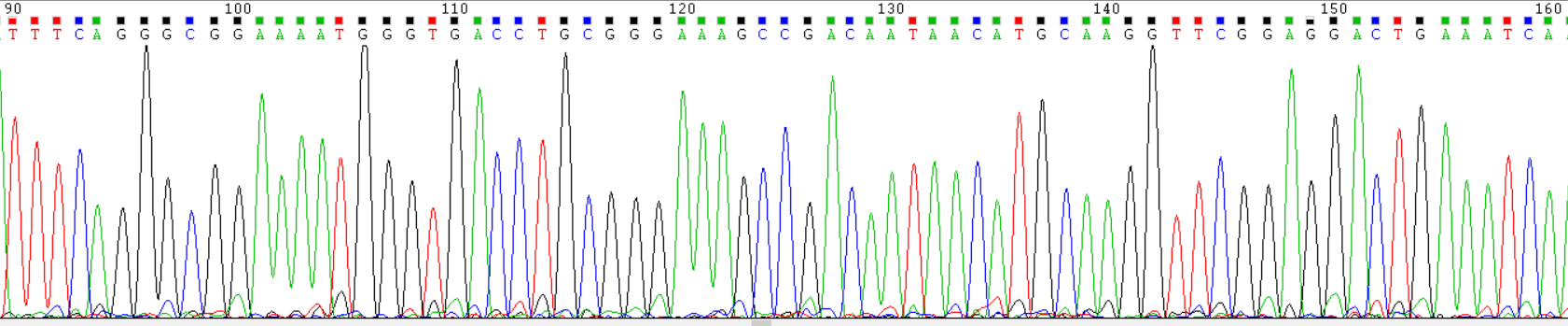

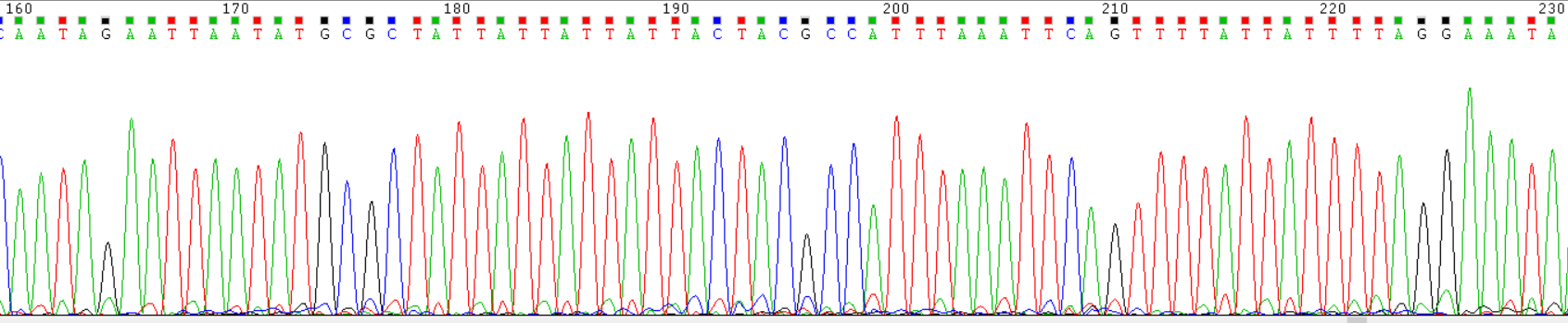

Supplement: S1 File — (DOCX) [file pone.0281297.s012.docx]
